# Ruthenium Complexes Containing Heterocyclic Thioamidates Trigger Caspase-Mediated Apoptosis Through MAPK Signaling in Human Hepatocellular Carcinoma Cells

**DOI:** 10.3389/fonc.2019.00562

**Published:** 2019-07-09

**Authors:** Sara P. Neves, Nanashara C. de Carvalho, Monize M. da Silva, Ana Carolina B. C. Rodrigues, Larissa M. Bomfim, Rosane B. Dias, Caroline B. S. Sales, Clarissa A. Gurgel Rocha, Milena B. P. Soares, Alzir A. Batista, Daniel P. Bezerra

**Affiliations:** ^1^Gonçalo Moniz Institute, Oswaldo Cruz Foundation (IGM-FIOCRUZ/BA), Salvador, Brazil; ^2^Department of Chemistry, Federal University of São Carlos, São Carlos, Brazil; ^3^Department of Biomorphology, Institute of Health Sciences, Federal University of Bahia, Salvador, Brazil

**Keywords:** ruthenium complexes, heterocyclic thioamidates, apoptosis, ERK1/2, HepG2

## Abstract

Herein, ruthenium complexes containing heterocyclic thioamidates [Ru(mmi)(bipy)(dppb)]PF_6_ (**1**), [Ru(tzdt)(bipy)(dppb)]PF_6_ (**2**), [Ru(dmp)(bipy)(dppb)]PF_6_ (**3**) and [Ru(mpca)(bipy)(dppb)]PF_6_ (**4**) were investigated for their cellular and molecular effects in cancer cell lines. Complexes **1** and **2** were the most potent of the four compounds against a panel of different cancer cell lines in monolayer cultures and showed potent cytotoxicity in a 3D model of multicellular spheroids that formed from human hepatocellular carcinoma HepG2 cells. In addition, both complexes were able to bind to DNA in a calf thymus DNA model. Compared to the controls, a reduction in cell proliferation, phosphatidylserine externalization, internucleosomal DNA fragmentation, and the loss of the mitochondrial transmembrane potential were observed in HepG2 cells that were treated with these complexes. Additionally, coincubation with a pan-caspase inhibitor (Z-VAD(OMe)-FMK) reduced the levels of apoptosis that were induced by these compounds compared to those in the negative controls, indicating that cell death through apoptosis occurred via a caspase-dependent pathway. Moreover, these complexes also induced the phosphorylation of ERK1/2, and coincubation with an MEK inhibitor (U0126), which is known to inhibit the activation of ERK1/2, but not JNK/SAPK and p38 MAPK inhibitors, reduced the complexes-induced apoptosis compared to that in the negative controls, indicating that the induction of apoptotic cell death occurred through ERK1/2 signaling in HepG2 cells. On the other hand, no increase in oxidative stress was observed in HepG2 cells treated with the complexes, and the complexes-induced apoptosis was not reduced with coincubation with the antioxidant N-acetylcysteine or a p53 inhibitor compared to that in the negative controls, indicating that apoptosis occurred via oxidative stress- and p53-independent pathways. Finally, these complexes also reduced the growth of HepG2 cells that were engrafted in C.B-17 SCID mice compared to that in the negative controls. These results indicated that these complexes are novel anticancer drug candidates for liver cancer treatment.

## Introduction

Liver cancer is the sixth most common cancer and the fourth most common cause of cancer lethality worldwide. The estimated incidence of liver cancer in 2018 included approximately 841,080 new cases and 781,631 deaths, which represents an overall ratio of mortality to incidence of 0.93, indicating that the prognosis for liver cancer is very poor (1). Hepatocellular carcinoma (HCC) is the most common type of primary liver cancer, accounting for approximately 75% of cases. For advanced HCC, sorafenib, a tyrosine kinase inhibitor, is a validated systemic therapy, but this treatment prolongs survival by only 3 months (2–4). Therefore, new drugs to treat liver cancer are needed.

In regard to metallodrugs, platinum-based drugs are widely used in the treatment of a variety of cancers, and ruthenium-based drugs have emerged as a novel potential class of chemotherapeutic and antineoplastic drugs (5). Ruthenium-based drugs have been considered an attractive alternative to platinum-based drugs because some ruthenium complexes have been shown to be more selective for cancer cells. This property has been attributed to the ability of ruthenium to mimic iron in binding to several biological molecules, including serum proteins (e.g., transferrin and albumin) (6). Therefore, many ruthenium complexes have been synthesized as novel anticancer agents (7–13). In particular, the ruthenium complexes [ImH]trans-[RuCl_4_(Im)(dmso-S)] (NAMI-A, where Im = imidazole) and [IndH]trans-[RuCl_4_(Ind)_2_] (KP1019, where Ind = indazole) completed phase I/II clinical trials in 2004 and 2008, respectively (14, 15). NAMI-A has been reported to interfere with the regulation of cell cycle and extracellular matrix invasion, thus preventing tumor metastasis, and KP1019 causes cell death through apoptosis via the formation of reactive oxygen species and the activation of the intrinsic mitochondrial pathway (16, 17).

**Figure 1:**
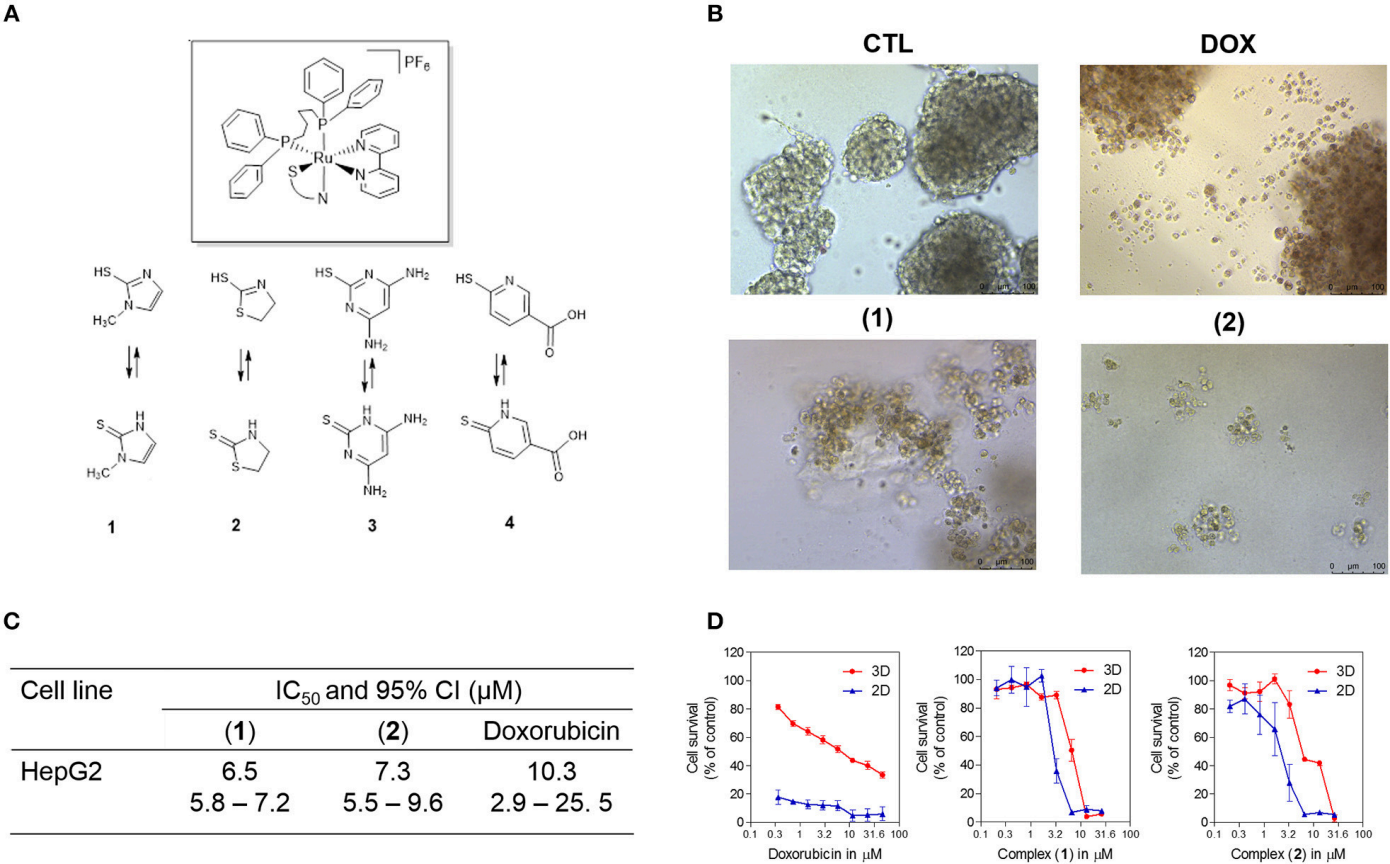
Effect of ruthenium complexes containing heterocyclic thioamidates in a 3D *in vitro* model of cancer multicellular spheroids formed from HepG2 cells. **(A)** Chemical structure of the ruthenium complexes containing heterocyclic thioamidates. **(B)** Cells were examined by light microscopy (bar = 100 μm) at the highest concentration tested after 72 h of incubation. **(C)** IC_50_ values and their respective 95% confidence intervals (95% CI) in μM. **(D)** Cell survival curves of the 3D vs. 2D culture models. The curves were obtained with non-linear regression from at least three independent experiments that were performed in duplicate and were measured with an Alamar blue assay after 72 h of incubation. The negative control (CTL) was treated with the vehicle (0.5% DMSO) that was used to solubilize and dilute the complexes, and doxorubicin (DOX) was used as the positive control.

Recently, ruthenium complexes containing heterocyclic thioamidates [Ru(mmi)(bipy)(dppb)]PF_6_ (**1**), [Ru(tzdt)(bipy)(dppb)]PF_6_ (**2**), [Ru(dmp)(bipy)(dppb)]PF_6_ (**3**) and [Ru(mpca)(bipy)(dppb)]PF_6_ (**4**) [where mmi = mercapto-1-methyl-imidazole; tzdt = 1,3-thiazolidine-2-thione; dmp = 4,6-diamino-2-mercaptopyrimidine; mpca = 6-mercaptopyridine-3-carboxylic acid; bipy = 2,2′-bipyridine; and dppb = 1,4-bis(diphenylphosphino)butane] (Figure 1A) were synthesized and shown to have potent cytotoxicity in cancer cell lines, to be able to bind to DNA and to inhibit the supercoiled DNA relaxation that is mediated by human topoisomerase IB (18, 19). However, cellular and molecular mechanisms of these compounds in causing cancer cell cytotoxicity have not been extensively explored. In the present work, these ruthenium complexes were investigated for their underlying cellular and molecular mechanisms in cancer cell cytotoxicity.

## Materials and Methods

### Synthesis of Ruthenium Complexes Containing Heterocyclic Thioamidates

The ruthenium complexes containing heterocyclic thioamidates [Ru(mmi)(bipy)(dppb)]PF_6_ (**1**), [Ru(tzdt)(bipy)(dppb)]PF_6_ (**2**), [Ru(dmp)(bipy)(dppb)]PF_6_ (**3**) and [Ru(mpca)(bipy)(dppb)]PF_6_ (**4**) were synthesized as previously reported (18, 19). Briefly, the complexes were prepared by reacting the *cis*-[RuCl_2_(dppb)(bipy)] precursor (0.132 mmol) with the ligands (0.150 mmol) and KPF_6_ (0.132 mmol) in methanol (50 mL) in an Ar atmosphere for 24 h. The final orange solution was concentrated to approximately 2 mL, and diethyl ether was added to obtain an orange precipitate. The solid was filtered, well rinsed with water (5 × 5 mL) and diethyl ether (3 × 5 mL) and dried in vacuo. All manipulations were performed under argon. All reagents were purchased from Sigma-Aldrich (Sigma-Aldrich Co., Saint Louis, MO, USA) and were used as received.

## *In vitro* Assays

### Cells

The cell lines HepG2 (human hepatocellular carcinoma), HL-60 (human promyelocytic leukemia), K-562 (human chronic myelogenous leukemia), B16-F10 (mouse melanoma), MCF-7 (human breast carcinoma), HCT116 (human colon carcinoma), HSC-3 (human oral squamous cell carcinoma), SCC-4 (human oral squamous cell carcinoma), MRC-5 (human lung fibroblast), WT SV40 MEF (wild-type immortalized mouse embryonic fibroblast), and BAD KO SV40 MEF (BAD gene knockout immortalized mouse embryonic fibroblasts) were obtained from the American Type Culture Collection (ATCC, Manassas, VA, USA). Cells were cultured in RPMI 1640 medium (Gibco-BRL, Gaithersburg, MD, USA) with 10% fetal bovine serum (Life, Carlsbad, CA, USA), 2 mM L-glutamine (Vetec Química Fina, Duque de Caxias, RJ, Brazil) and 50 μg/mL gentamycin (Life). For all experiments, adherent cells were collected by treatment with 0.25% trypsin-EDTA solution (Gibco-BRL). All cell lines were cultured in flasks at 37°C in 5% CO_2_ and were subcultured every 3–4 days to maintain exponential growth. All cell lines were tested for mycoplasma using a mycoplasma staining kit (Sigma-Aldrich Co.) to validate that the cells that were used were free from contamination.

Heparinized blood was collected from 20 to 35-year-old, non-smoking healthy donors who had not taken any drugs for at least 15 days prior to sampling, and peripheral blood mononuclear cells (PBMCs) were isolated using a Ficoll density gradient in GE Ficoll-Paque Plus (GE Healthcare Bio-Sciences AB, Sweden). PBMCs were washed and resuspended at a concentration of 3 × 10^5^ cells/mL in RPMI 1640 medium with 20% fetal bovine serum, 2 mM glutamine and 50 μg/mL gentamycin at 37°C with 5% CO_2_. Concanavalin A (ConA, Sigma-Aldrich Co.) was used as a mitogen to trigger cell division in T-lymphocytes. ConA (10 μg/mL) was added at the beginning of the culture and the cells were treated with the test compounds after 24 h. Cell viability was examined using a trypan blue exclusion assay for all experiments. The Research Ethics Committee of the Oswaldo Cruz Foundation (Salvador, Bahia, Brazil) approved the experimental protocol (#031019/2013). All participants signed a written informed consent form to participate in the study.

### Cytotoxicity Assay

Cytotoxicity was measured colorimetrically using an Alamar blue method that was performed as previously described (20, 21). Cells were seeded in 96-well plates for all experiments (7 × 10^4^ cells/mL for adherent cells or 3 × 10^5^ cells/mL for suspension cells in 100 μL of medium) and incubated overnight at 37°C with 5% CO_2_ to allow for adherence. The complexes were dissolved in 0.5% dimethyl sulfoxide (DMSO, Vetec Química Fina) at eight different concentrations from 0.19 to 25 μg/mL and were added to each well and incubated for 72 h. The negative control was treated with the vehicle (0.5% DMSO) that was used to solubilize and dilute the complexes. Doxorubicin (purity ≥ 95%, doxorubicin hydrochloride, Laboratory IMA S.A.I.C., Buenos Aires, Argentina) and oxaliplatin (Sigma-Aldrich Co.) were used as the positive controls. Four (for cell lines) or 24 h (for PBMCs) before the end of incubation, 20 μL of a stock solution (0.312 mg/mL) of resazurin (Sigma-Aldrich Co.) was added to each well. Absorbance was measured at 570 and 600 nm using a SpectraMax 190 Microplate Reader (Molecular Devices, Sunnyvale, CA, USA).

### 3D Multicellular Spheroid Culture

HepG2 cells were cultivated as 3D multicellular spheroids in a 96-well plate with a cell-repellent surface. Briefly, 100 μL of a suspension of cells (5 × 10^5^ cells/mL) was seeded into a 96-well plate with a cell-repellent surface (Greiner Bio-One, Kremsmünster, Austria) and cultured in RPMI 1640 medium with 10% fetal bovine serum, 2 mM glutamine plus 3% Matrigel (BD Biosciences, San Jose, CA, EUA) and 50 μg/mL gentamycin at 37°C with 5% CO_2_. Spheroids with stable structures formed after 3 days. Then, the complexes were dissolved in 0.5% DMSO at eight different concentrations from 0.19 to 25 μg/mL, were added to each well and incubated for 72 h. The negative control was treated with the vehicle (0.5% DMSO) that was used to solubilize and dilute the complexes, and doxorubicin was used as the positive control. At the end of the experiment, morphological changes were examined by light microscopy (Olympus BX41, Tokyo, Japan) using Image-Pro software (Media Cybernetics, Inc. Silver Spring, USA), and cytotoxicity was quantified with the Alamar blue method as described above.

### DNA Interaction Assay

DNA interaction was measured in a cell-free system as previously described (22). In this assay, the ability of the complexes to translocate ethidium bromide, a DNA intercalator, from calf thymus DNA (ctDNA, Sigma-Aldrich Co.) was examined. The assay was conducted in 96-well plates, and the reaction mixture contained 15 μg/mL ctDNA, 1.5 μM ethidium bromide (Sigma-Aldrich Co.) and 10 or 20 μM of complexes in 100 μL of saline solution. The negative control was treated with the vehicle (0.2% DMSO) that was used to solubilize and dilute the complexes, and doxorubicin was used as the positive control. After a 15 min incubation at room temperature, fluorescence was measured using the excitation and emission wavelengths of 320 and 600 nm, respectively, using a Spectramax Microplate Reader (Molecular Devices).

### Trypan Blue Exclusion Assay

For all subsequent *in vitro* experiments, 2 mL of a suspension of HepG2 cells were seeded (7 × 10^4^ cells/mL) and incubated in a 24-well plate overnight to allow the cells to adhere to the plate surface. Then, the cells were incubated with 5 and/or 10 μM of complex **1** and **2** and/or 4 μM of complex **2** for 24 and/or 48 h (or until an indicated time point). The negative control was treated with the vehicle (0.2% DMSO) that was used to solubilize and dilute the complexes, and doxorubicin (2 μM) was used as the positive control.

In the trypan blue exclusion assay, the number of viable cells and non-viable cells (stained with trypan blue) were counted. Briefly, 90 μL was removed from the cell suspension, and 10 μL of trypan blue (0.4%) was added. Cell counting was performed using a light microscope with a hemocytometer filled with an aliquot of the homogenized cell suspension.

### May-Grunwald-Giemsa Staining

To evaluate alterations in the morphology, cells were cultured on a coverslip and stained with May-Grunwald-Giemsa. Morphological changes were examined by light microscopy (Olympus BX41) using Image-Pro software (Media Cybernetics).

### Flow Cytometric Assays

Internucleosomal DNA fragmentation and cell cycle distribution were measured by the quantification of the DNA content (23). In this assay, cells were harvested in a permeabilization solution containing 0.1% Triton X-100, 2 μg/mL propidium iodide, 0.1% sodium citrate and 100 μg/mL RNAse (all from Sigma-Aldrich Co.) and incubated in the dark for 15 min at room temperature.

A FITC Annexin V Apoptosis Detection Kit I (BD Biosciences) was used for apoptosis quantification, and the analysis was performed according to the manufacturer's instructions. Cell fluorescence and light scattering features were determined by flow cytometry. The percentages of viable, early apoptotic, late apoptotic and necrotic cells were determined. Protection assays using a pan-caspase inhibitor (Z-VAD(Ome)-FMK, Cayman Chemical; Ann Arbor, MI, USA), the antioxidant *N*-acetylcysteine (NAC, Sigma-Aldrich Co.), a Jun kinase (JNK/SAPK) inhibitor (SP600125; Cayman Chemical), a p38 MAPK inhibitor (PD169316; Cayman Chemical), a MEK (mitogen-activated protein kinase kinase) inhibitor (U0126; Cayman Chemical), and a p53 inhibitor (cyclic pifithrin-α; Cayman Chemical) were also performed. In these assays, the cells were preincubated for 2 h with 50 μM Z-VAD(Ome)-FMK, 5 μM SP600125, 5 μM PD169316, 5 μM U0126 or 10 μM cyclic pifithrin-α, followed by incubation with the complexes at an established concentration (10 μM for complex **1** and 4 μM for complex **2**) for 48 h.

The mitochondrial transmembrane potential was evaluated by the retention of the rhodamine 123 dye (24). Cells were incubated with rhodamine 123 (5 μg/mL, Sigma-Aldrich Co.) at 37°C for 15 min in the dark and washed with saline. The cells were then incubated again in saline at 37°C for 30 min in the dark, and the cell fluorescence was determined by flow cytometry.

The reactive oxygen species (ROS) levels were measured using 2′,7′-dichlorofluorescin diacetate (DCF-DA) (Sigma-Aldrich Co.) (25). The cells were treated with the complexes for 1 or 3 h. Then, the cells were collected, washed with saline and resuspended in tubes with saline containing 5 μM DCF-DA for 30 min. Finally, the cells were washed with saline, and cell fluorescence was determined by flow cytometry.

Phosphorylated extracellular signal-regulated kinase (ERK), JNK/SAPK and p38 MAPK were quantified by flow cytometry as previously described (26). The cells were collected and resuspended in 0.5–1 mL of 4% formaldehyde and fixed for 10 min at 37°C. Then, the tubes were chilled on ice for 1 min. The cells were permeabilized by slowly adding ice-cold 100% methanol to the cooled cells while gently vortexing to a final concentration of 90% methanol and incubating for 30 min on ice. After washing with incubation buffer (0.5% bovine serum albumin in PBS), PE mouse anti-JNK/SAPK (pT183/pY185) (ID 562480), PE mouse anti-p38 MAPK (pT180/pY182) (ID 612565), PE mouse anti-ERK1/2 (pT202/pY204) (ID 612566) or PE mouse IgG_1_,κ isotype control (ID 555749) antibodies, all from BD Biosciences, were added and incubated for 1 h at room temperature. Finally, the cells were washed with PBS, and the cell fluorescence was measured by flow cytometry.

For all flow cytometry analyses, at least 10^4^ events were recorded per sample using a BD LSRFortessa cytometer, BD FACSDiva Software (BD Biosciences) and FlowJo Software 10 (FlowJo Lcc; Ashland, OR, USA). Cellular debris was omitted from the analysis.

## *In vivo* assays

### Animals

A total of 64 C.B*-*17 severe-combined immunodeficiency (SCID) mice (females, 25–30 g) were obtained and maintained at the animal facilities from Gonçalo Moniz Institute-FIOCRUZ (Salvador, Bahia, Brazil). Animals were housed in cages with free access to food and water. All animals were kept under a 12:12-h light-dark cycle (lights on at 6:00 a.m.). The animals were treated according to the ethical principles for animal experimentation of SBCAL (Brazilian Association of Laboratory Animal Science), Brazil. The Animal Ethics Committee of Gonçalo Moniz Institute–FIOCRUZ (Salvador, Bahia, Brazil) approved the experimental protocol (number 06/2015). Animal welfare was monitored throughout the study, and the pain and suffering were minimized.

### Human Hepatocellular Carcinoma Xenograft Model

A human hepatocellular carcinoma xenograft model was generated by subcutaneously implanting HepG2 cells (1 × 10^7^ cells per 500 μL) into the left front armpit of C.B*-*17 SCID mice. At the beginning of the experiment, the mice were randomly divided into six groups: group 1 animals received injections of vehicle with 5% DMSO solution (*n* = 10); group 2 animals received injections of doxorubicin (0.3 mg/kg/day, *n* = 8); group 3 animals received injections of complex **1** (0.5 mg/kg/day, *n* = 10); group 4 animals received injections of complex **1** (1 mg/kg/day, *n* = 10); group 5 animals received injections of complex **2** (0.5 mg/kg/day, *n* = 10); and group 6 animals received injections of complex **2** (1 mg/kg/day, *n* = 10). The treatments were initiated one day after the cancer cell injection. The animals were treated intraperitoneally (200 μL per animal) once a day for 21 consecutive days. On the 22nd day, the animals were anesthetized, and peripheral blood samples were collected from the brachial artery. Animals were euthanized by an anesthetic overdose, and tumors were excised and weighed.

### Toxicological Evaluation

To evaluate the toxicological aspects, mice were weighed at the beginning and at the end of the experiment. Animals were observed for signs of abnormalities throughout the study. Hematological analysis was performed using an Advia 60 hematology system (Bayer, Leverkusen, Germany). Before fixation in 4% formaldehyde, tumors, livers, kidneys, hearts, and lungs were examined for size, color, and hemorrhaging. Histological analyses were performed under optical microscopy using hematoxylin/eosin and periodic acid-schiff (liver) staining by an experienced pathologist.

### Statistical Analysis

Data are presented as the means ± S.E.M. or as the half-maximal inhibitory concentration (IC_50_) values with 95% confidence intervals, which were obtained by non-linear regression. Differences among experimental groups were compared using analysis of variance (ANOVA) followed by the Student–Newman–Keuls test (*P* < 0.05). All statistical analyses were performed using GraphPad Prism (Intuitive Software for Science; San Diego, CA, USA).

## Results

### Ruthenium Complexes Containing Heterocyclic Thioamidates Exhibit Potent Cytotoxicity to Different Cancer Cells

The cytotoxic potential of ruthenium complexes containing heterocyclic thioamidates was evaluated in eight different cancer cell lines (HepG2, HL-60, K-562, B16-F10, MCF-7, HCT116, HSC-3, and SCC-4) and in two non-cancerous cell lines (PBMCs and MRC-5) with the Alamar blue method after 72 h of incubation. Table 1 shows the results that were obtained. Complexes **1** and **2** were the most potent cytotoxic agents. Complex **1** had IC_50_ values that ranged from 1.4 to 2.6 μM for the cancer cell lines K-562 and HepG2, respectively; complex **2** had IC_50_ values that ranged from 0.9 to 7.3 μM for the cancer cell lines K-562 and MCF-7, respectively; complex **3** had IC_50_ values that ranged from 3.9 to 8.7 μM for the cancer cell lines B16-F10 and MCF-7, respectively; and complex **4** had IC_50_ values that ranged from 8.7 to 17.7 μM for the cancer cell lines HL-60 and B16-F10, respectively. Doxorubicin had IC_50_ values that ranged from 0.2 to 4 μM for the cancer cell lines HL-60/B16-F10 and SCC-4, respectively, while oxaliplatin had IC_50_ values that ranged from 0.6 to 7.7 μM for the cancer cell lines HL-60 and SCC-4, respectively. The selectivity index (SI) of each complex was calculated using the following formula: SI = IC_50_ [noncancerous cells]/IC_50_ [cancer cells]. Table 2 shows the calculated selectivity index. For most cancer cell lines, the complexes exhibited a selectivity index similar to that of the positive controls doxorubicin and oxaliplatin. Since complexes **1** and **2** were the most potent cytotoxic agents, these complexes were selected for further experiments.

**Table 1:** Cytotoxic activity of ruthenium complexes containing heterocyclic thioamidates.

**Cells**	**IC**_****50****_ **and 95% CI (μM)**					
	**(1)**	**(2)**	**(3)**	**(4)**	**DOX**	**OXA**
**CANCER CELLS**						
HepG2	2.6 1.8–3.7	1.9 1.4–2.7	7.2 6.3–8.1	14.2 12.3–16.5	0.3 0.2–0.4	2.2 1.3–3.8
HL-60	2.0 1.3–3.2	1.1 0.6–1.9	5.0 3.4–7.3	8.7 7.4–10.1	0.2 0.1–0.3	0.6 0.1–0.8
K-562	1.4 1.1–1.8	0.9 0.6–1.2	4.6 3.4–6.3	12.8 11.0–14.9	0.9 0.8–1.2	1.0 0.1–1.3
B16-F10	1.8 1.0–3.2	1.3 0.8–2.3	3.9 2.7–5.6	17.7 16.0–19.6	0.2 0.1–0.3	2.2 1.2–4.1
MCF-7	N.d.	7.3 5.6–9.4	8.7 7.8–9.7	N.d.	0.4 0.3–0.5	5.9 3.5–9.9
HCT116	N.d.	2.7 1.6–4.4	5.9 5.0–6.8	N.d.	0.3 0.2–0.4	4.1 2.3–5.5
HSC-3	N.d.	2.2 1.0–4.8	5.2 4.2–6.4	N.d.	0.5 0.4–0.7	3.3 1.4–7.8
SCC-4	N.d.	3.9 2.5–6.1	4.7 2.9–7.6	N.d.	4.0 2.0–8.2	7.7 4.6–13.0
**NONCANCEROUS CELLS**						
PBMCs	2.7 2.4–3.1	2.6 2.3–3.0	12.9 10.6–15.8	22.5 14.9–33.9	2.5 2.3–2.6	9.4 6.5 - 11.4
MRC-5	2.3 1.6–3.4	1.6 1.1–2.1	5.3 4.7–5.9	N.d.	0.4 1.2–0.4	1.5 0.9–2.9

*Data are presented as the IC_50_ values and 95% confidence intervals (95% CI) in μM that were obtained with non-linear regression from at least three independent experiments that were performed in duplicate, as measured by an Alamar blue assay after 72 h of incubation. Cancer cells: HepG2 (human hepatocellular carcinoma); HL-60 (human acute promyelocytic leukemia); K-562 (human chronic myelogenous leukemia); B16-F10 (mouse melanoma); MCF-7 (human breast adenocarcinoma); HCT116 (human colon carcinoma); HSC-3 (human oral squamous cell carcinoma); and SCC-4 (human oral squamous cell carcinoma). Non-cancerous cells: PBMCs (human peripheral blood mononuclear cells) and MRC-5 (human lung fibroblasts). Doxorubicin (DOX) and oxaliplatin (OXA) were used as the positive controls. N.d. = Not determined*.

**Table 2:** Selectivity index of ruthenium complexes containing heterocyclic thioamidates.

**Cancer cells**	**Noncancerous cells**											
	**PBMCs**						**MRC-5**					
	**(1)**	**(2)**	**(3)**	**(4)**	**DOX**	**OXA**	**(1)**	**(2)**	**(3)**	**(4)**	**DOX**	**OXA**
HepG2	1.0	1.4	1.8	1.6	8.3	4.3	0.9	0.8	0.7	N.d.	1.3	0.7
HL-60	1.4	2.4	2.6	2.6	12.5	15.7	1.2	1.5	1.1	N.d.	2.0	2.5
K-562	1.9	2.9	2.8	1.8	2.8	9.4	1.6	1.8	1.2	N.d.	0.4	1.5
B16-F10	1.5	2.0	3.3	1.3	12.5	4.3	1.3	1.2	1.4	N.d.	2.0	0.7
MCF-7	N.d.	0.4	1.5	N.d.	6.3	1.6	N.d.	0.2	0.6	N.d.	1.0	0.3
HCT116	N.d.	1.0	2.2	N.d.	8.3	2.3	N.d.	0.6	0.9	N.d.	1.3	0.4
HSC-3	N.d.	1.2	2.5	N.d.	5.0	2.9	N.d.	0.7	1.0	N.d.	0.8	0.5
SCC-4	N.d.	0.7	2.8	N.d.	0.6	1.2	N.d.	0.4	1.1	N.d.	0.1	0.2

*The selectivity index (SI) of each complex was calculated using the following formula: SI = IC_50_ [noncancerous cells]/IC_50_ [cancer cells]. Cancer cells: HepG2 (human hepatocellular carcinoma); HL-60 (human acute promyelocytic leukemia); K-562 (human chronic myelogenous leukemia); B16-F10 (mouse melanoma); MCF-7 (human breast adenocarcinoma); HCT116 (human colon carcinoma); HSC-3 (human oral squamous cell carcinoma); and SCC-4 (human oral squamous cell carcinoma). Noncancerous cells: PBMCs (human peripheral blood mononuclear cells) and MRC-5 (human lung fibroblast). Doxorubicin (DOX) and oxaliplatin (OXA) were used as the positive controls. N.d. = Not determined*.

Next, the cytotoxicity of complexes **1** and **2** were evaluated in an *in vitro* 3D model with cancer multicellular spheroids that formed from HepG2 cells, by using the Alamar blue method after 72 h of incubation. This cell line was chosen as a cellular model because it was among the most sensitive cell lines to the complexes. After treatment with both complexes, the cells in the spheroids were dissociated, indicating that drug penetrance and cytotoxicity had occurred in the 3D model (Figure 1B). The IC_50_ values were 6.5 and 7.3 μM for complexes **1** and **2**, respectively (Figure 1C). Doxorubicin had an IC_50_ value of 10.3 μM. Figure 1D shows the cell survival curves of 3D vs. 2D culture models for these complexes.

**Figure 2:**
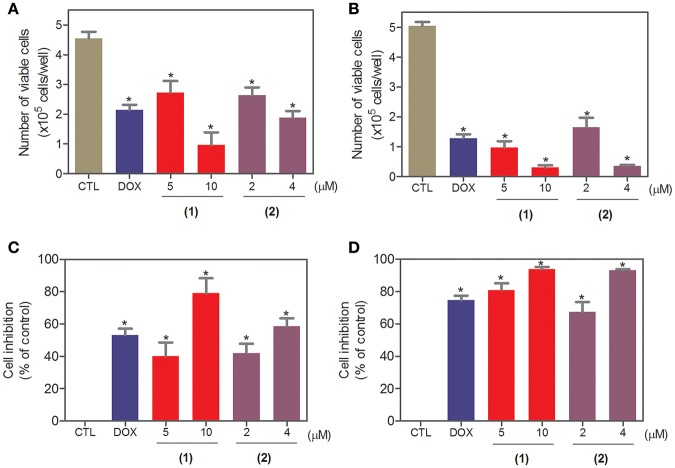
Effect of ruthenium complexes containing heterocyclic thioamidates on the cell viability of HepG2 cells, as determined by the trypan blue exclusion method after 24 **(A,C)** and 48 **(B,D)** h of incubation. The negative control (CTL) was treated with the vehicle (0.2% DMSO) that was used to solubilize and dilute the complexes, and doxorubicin (DOX, 2 μM) was used as the positive control. Data are presented as the mean ± S.E.M. of at least three independent experiments that were performed in duplicate. *P < 0.05 compared with the negative control, as determined with ANOVA followed by the Student-Newman-Keuls test.

To complement the Alamar blue method, the cell viability was evaluated after 24 and 48 h of treatment with complexes **1** and **2** with the trypan blue exclusion method in HepG2 cells. Both complexes significantly reduced (*p* < 0.05) the number of viable cells (Figure 2) compared to that of the negative controls. At concentrations of 5 and 10 μM, complex **1** reduced the number of viable cells by 40.1 and 78.9% after 24 h and 80.9 and 93.9% after 48 h, respectively, compared to that of the negative controls. At concentrations of 2 and 4 μM, complex **2** reduced the number of viable cells by 42.1 and 58.6% after 24 h and 67.3 and 93.1% after 48 h, respectively, compared to that of the negative controls. None of the complexes induced a significant (*p* > 0.05) increase in the number of non-viable cells compared to that in the negative controls. Doxorubicin also reduced the number of viable cells by 53.1 and 74.7% after 24 and 48 h of incubation, respectively, compared to that of the negative controls.

### Ruthenium Complexes Containing Heterocyclic Thioamidates Interact With DNA

**Figure 3:**
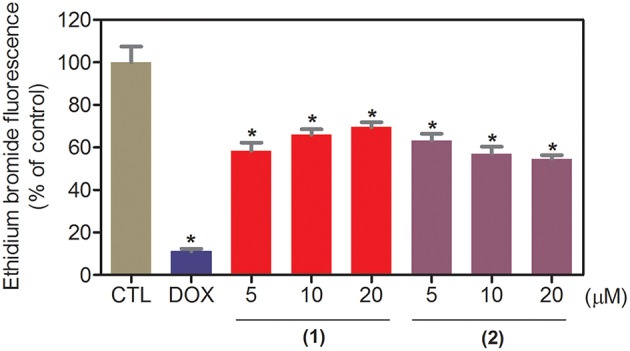
DNA interaction of ruthenium complexes containing heterocyclic thioamidates, as evaluated by the ability of the complexes to displace ethidium bromide from calf thymus DNA. The negative control (CTL) was treated with the vehicle (0.2% DMSO) that was used to solubilize and dilute the complexes, and doxorubicin (DOX, 20 μM) was used as the positive control. Data are presented as the mean ± S.E.M. of three independent experiments that were performed in duplicate. *P < 0.05 compared with the negative control, as determined by ANOVA, followed by the Student-Newman-Keuls test.

The DNA-binding ability of ruthenium complexes containing heterocyclic thioamidates was assessed in a cell-free system using a ctDNA model. In this assay, the ability of the drugs to intercalate with the DNA is quantified by an ethidium bromide replacement method, where the drugs displace intercalated ethidium bromide from ctDNA and subsequently reduce the fluorescence of the ethidium bromide-DNA complex by releasing ethidium bromide in the solution. Both complexes significantly reduced (*p* < 0.05) the fluorescence intensity compared to that of the negative controls, indicating that both complexes are DNA intercalators (Figure 3). Compared to the negative controls, at concentrations of 5, 10, and 20 μM, complex **1** reduced the fluorescence intensity by 41.6, 34.0, and 30.4%, respectively, while complex **2** reduced the fluorescence intensity by 36.9, 42.9, and 45.4%, respectively, at the same concentrations. Doxorubicin, a known DNA intercalator, was used at 20 μM as the positive control, and reduced the fluorescence intensity by 89.0% compared to that of the negative controls.

### Ruthenium Complexes Containing Heterocyclic Thioamidates Trigger Caspase-Mediated Apoptosis in HepG2 Cells

**Figure 4:**
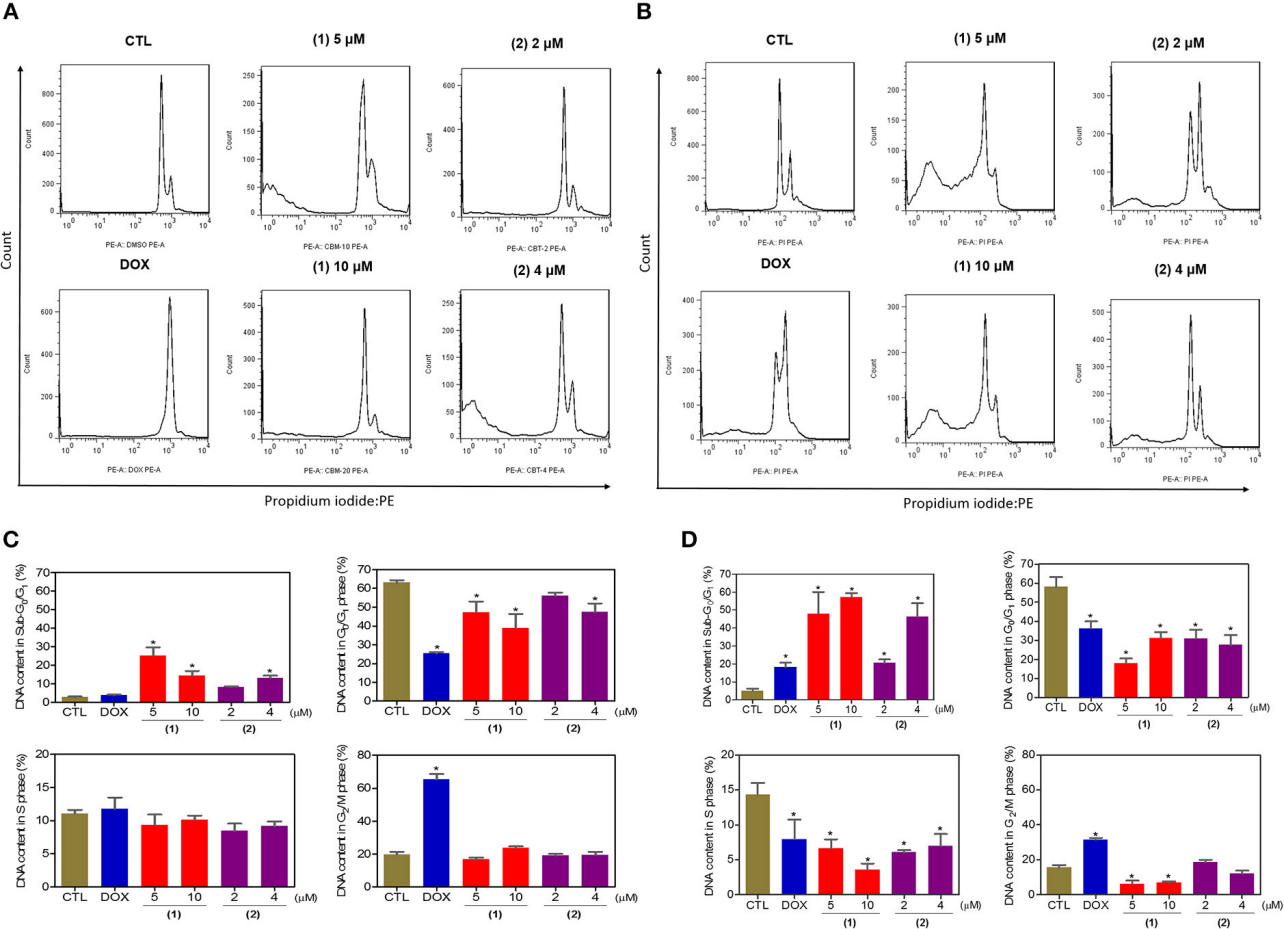
Effect of ruthenium complexes containing heterocyclic thioamidates on the cell cycle distribution of HepG2 cells after 24 **(A,C)** and 48 **(B,D)** h of incubation. The negative control (CTL) was treated with the vehicle (0.2% DMSO) that was used to solubilize and dilute the complexes, and doxorubicin (DOX, 2 μM) was used as the positive control. Data are presented as the mean ± S.E.M. of three independent experiments that were performed in duplicate. *P < 0.05 compared with the negative control, as determined by ANOVA, followed by the Student-Newman-Keuls test. Ten thousand events were evaluated per experiment, and cellular debris was omitted from the analysis.

Next, the DNA content was quantified by flow cytometry to measure the internucleosomal DNA fragmentation and cell cycle distribution in HepG2 cells treated with ruthenium complexes containing heterocyclic thioamidates after 24 and 48 h of incubation, and the results are shown in Figure 4. All DNA that was subdiploid (sub-G_0_/G_1_) was considered fragmented. Both complexes caused significant DNA fragmentation (*p* < 0.05) compared to that in the negative controls. Doxorubicin caused cell cycle arrest at the G_2_/M phase, followed by DNA fragmentation.

**Figure 5:**
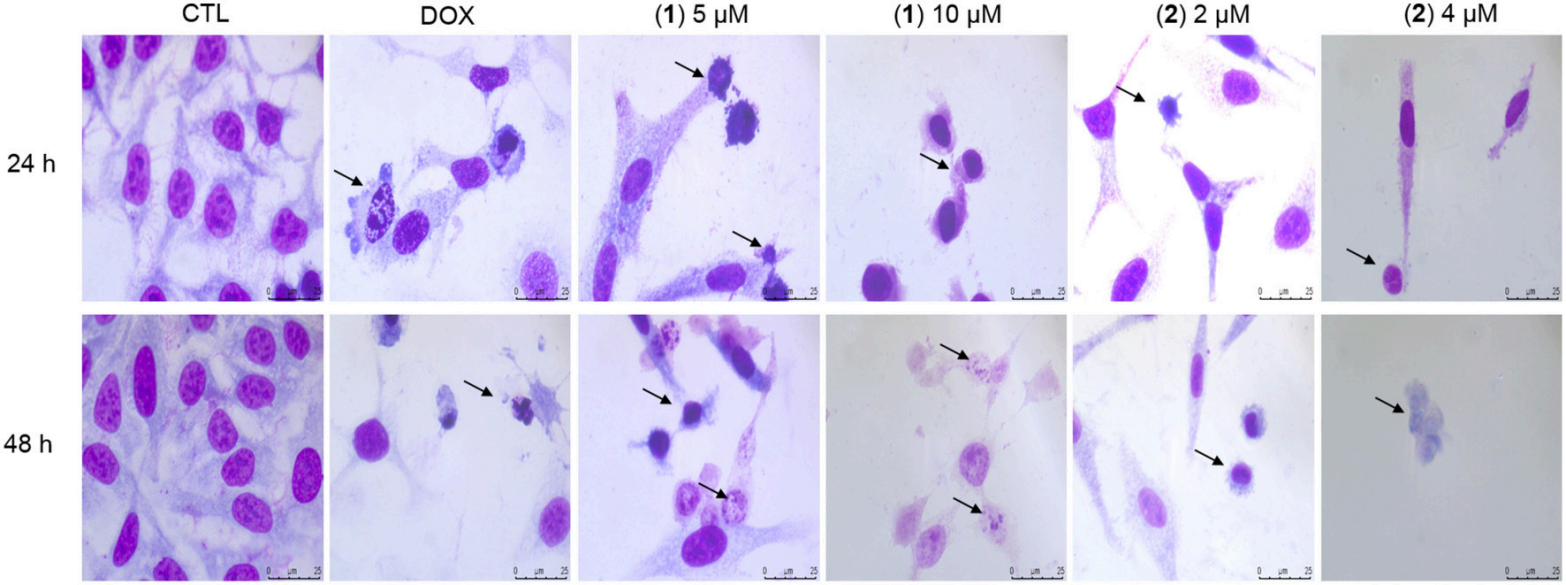
Effect of ruthenium complexes containing heterocyclic thioamidates in the morphological analysis of HepG2 cells after 24 and 48 h of incubation. The cells were stained with May-Grunwald-Giemsa and examined by light microscopy (bar = 20 μm). The negative control (CTL) was treated with the vehicle (0.2% DMSO) that was used to solubilize and dilute the complexes, and doxorubicin (DOX, 2 μM) was used as the positive control. Arrows indicate cell shrinkage or cells with fragmented DNA.

**Figure 6:**
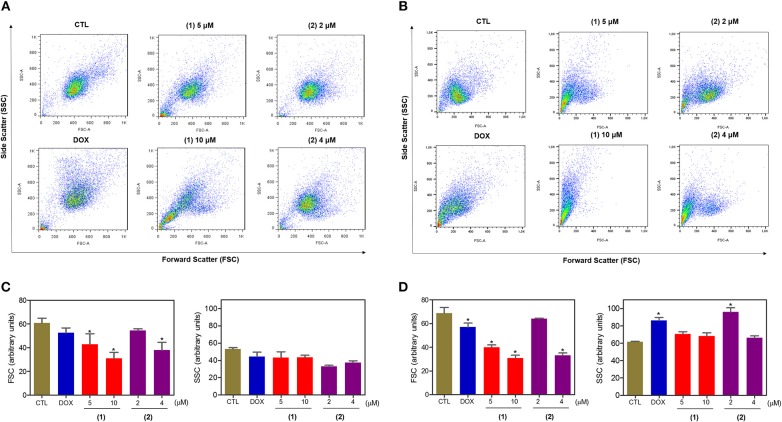
Effect of ruthenium complexes containing heterocyclic thioamidates on the morphology of HepG2 cells, as determined by light-scattering features detected by flow cytometry after 24 **(A,C)** and 48 **(B,D)** h of incubation. The negative control (CTL) was treated with the vehicle (0.2% DMSO) that was used to solubilize and dilute the complexes, and doxorubicin (DOX, 2 μM) was used as the positive control. Data are presented as the mean ± S.E.M. of three independent experiments that were performed in duplicate. *P < 0.05 compared with the negative control, as determined by ANOVA followed by the Student-Newman-Keuls test. Ten thousand events were evaluated per experiment, and cellular debris was omitted from the analysis.

Cell morphology was assessed by light microscopy using May-Grunwald-Giemsa staining after 24 and 48 h of incubation (Figure 5). Compared to the negative controls, both complexes induced a reduction in the cell volume, chromatin condensation and fragmentation of the nuclei, which are morphological aspects that are associated with cell death via apoptosis. To complement the light microscopy analysis, light-scattering features were assessed by flow cytometry and compared to the negative controls, both complexes caused cell shrinkage, as observed by the decrease in forward-light scatter, and nuclear condensation, as observed by the increase in side scatter (Figure 6). Doxorubicin also caused apoptosis-related morphological alterations.

**Figure 7:**
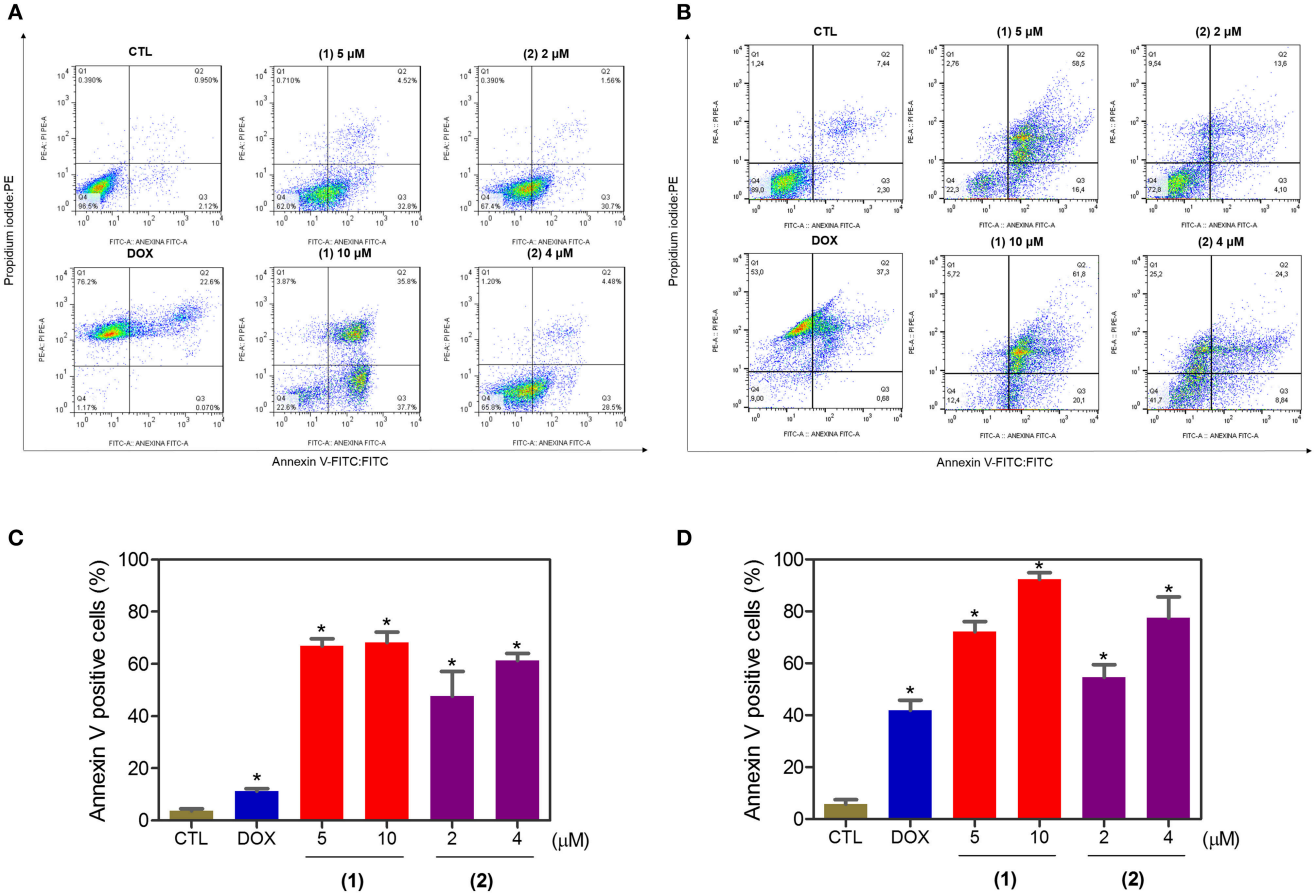
Effect of ruthenium complexes containing heterocyclic thioamidates on the induction of apoptosis in HepG2 cells, as determined by flow cytometry using annexin V-FITC/PI staining after 24 **(A,C)** and 48 **(B,D)** h of incubation. The negative control (CTL) was treated with the vehicle (0.2% DMSO) that was used to solubilize and dilute the complexes, and doxorubicin (DOX, 2 μM) was used as the positive control. Data are presented as the mean ± S.E.M. of three independent experiments that were performed in duplicate. *P < 0.05 compared with the negative control, as determined by ANOVA followed by the Student-Newman-Keuls test. Ten thousand events were evaluated per experiment, and cellular debris was omitted from the analysis.

**Figure 8:**
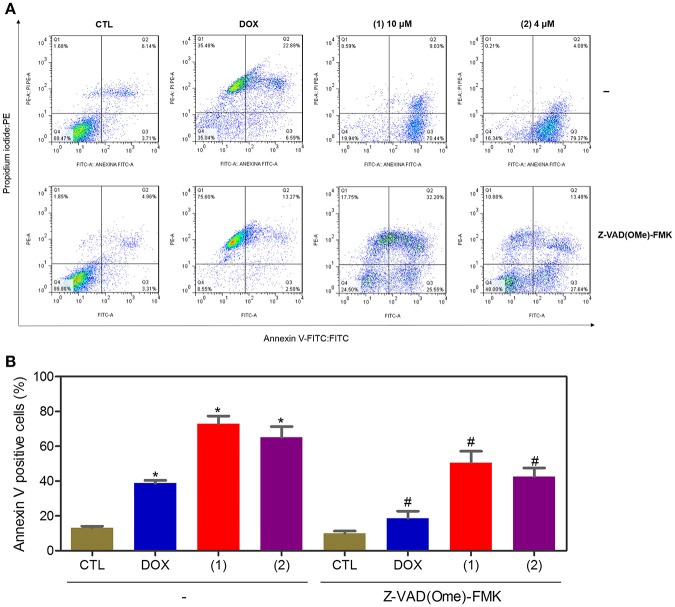
Effect of the pan-caspase inhibitor [Z-VAD(OMe)-FMK] on the apoptosis that was induced by ruthenium complexes containing heterocyclic thioamidates, as determined by flow cytometry using annexin V-FITC/PI staining. **(A)** Representative flow cytometry dot plots showing the percentage of cells in the viable, early-apoptotic, late-apoptotic, and necrotic stages. **(B)** Quantification of the apoptotic cells (early- and late-apoptotic cells). For the protection assay, the cells were preincubated for 2 h with 50 μM Z-VAD(Ome)-FMK and were then incubated with 10 μM of complex **1** or 4 μM of complex **2** for 48 h. The negative control (CTL) was treated with the vehicle (0.2% DMSO) that was used to solubilize and dilute the complexes, and doxorubicin (DOX, 2 μM) was used as the positive control. Data are presented as the mean ± S.E.M. of three independent experiments that were performed in duplicate. *P < 0.05 compared with the negative control, as determined by ANOVA followed by the Student-Newman-Keuls test. #*P* < 0.05 compared with the respective treatment without inhibitor, as determined by ANOVA followed by the Student-Newman-Keuls test. Ten thousand events were evaluated per experiment, and cellular debris was omitted from the analysis.

**Figure 9:**
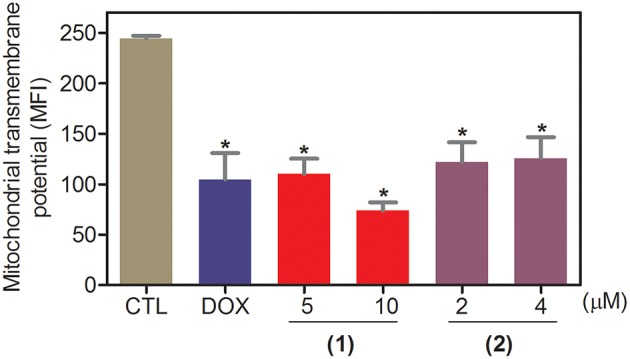
Effect of ruthenium complexes containing heterocyclic thioamidates on the mitochondrial membrane potential of HepG2 cells, as determined by flow cytometry using rhodamine 123 staining after 24 h of incubation. The negative control (CTL) was treated with the vehicle (0.2% DMSO) that was used to solubilize and dilute the complexes, and doxorubicin (DOX, 2 μM) was used as the positive control. Data are presented as the mean ± S.E.M. of three independent experiments that were performed in duplicate. *P < 0.05 compared with the negative control, as determined by ANOVA followed by the Student-Newman-Keuls test. Ten thousand events were evaluated per experiment, and cellular debris was omitted from the analysis. MFI: mean fluorescence intensity.

Apoptosis quantification was performed with annexin-V/PI double staining using flow cytometry after 24 and 48 h of incubation (Figure 7). Annexin-V positive cells were used as indicators of apoptosis. Compared to the negative controls, both complexes significantly increased the percentage of apoptotic cells at both incubation times (*P* < 0.05). No significant increase in the number of necrotic cells was observed in HepG2 cells treated with the complexes compared to that in the negative controls (*P* > 0.05). Furthermore, compared to the negative controls, coincubation with Z-VAD(OMe)-FMK, a pan-caspase inhibitor, partly reduced the apoptosis induced by both complexes (Figure 8), and the mitochondrial transmembrane potential was reduced after the treatment of HepG2 cells with these complexes (Figure 9), indicating that ruthenium complexes containing heterocyclic thioamidates induce cell death by apoptosis via caspase-dependent pathways. On the other hand, since coincubation with Z-VAD(OMe)-FMK did not fully block the complexes-induced apoptotic cell death, other cell death pathways, e.g., necroptosis and autophagy, may also be involved in the cell death that was caused by these complexes.

In complement to the effect of these complexes in cancer cells, the cytotoxicity of the complexes was also determined in the BAD KO SV40 MEF (BAD gene knockout immortalized mouse embryonic fibroblast) cell line and in its parental WT SV40 MEF (wild-type immortalized mouse embryonic fibroblast) cell line with the Alamar blue method after 72 h of incubation (Table S1). The IC_50_ values for complexes **1** and **2** were 0.7 and 2.9 μM for the BAD KO SV40 MEF cell line and 1.1 and 2.0 μM for the WT SV40 MEF cell line, respectively, indicating that the BAD gene is not essential for the cytotoxicity of these complexes in fibroblasts cells. Doxorubicin had IC_50_ values of 0.4 and 0.04 μM in the BAD KO SV40 MEF and WT SV40 MEF cell lines, respectively, while cisplatin had IC_50_ values of 47.3 and 36.9 μM in the BAD KO SV40 MEF and WT SV40 MEF cell lines, respectively.

### Ruthenium Complexes Containing Heterocyclic Thioamidates Cause ERK1/2-Mediated Apoptosis in HepG2 Cells Through ROS- and p53-Independent Pathways

**Figure 10:**
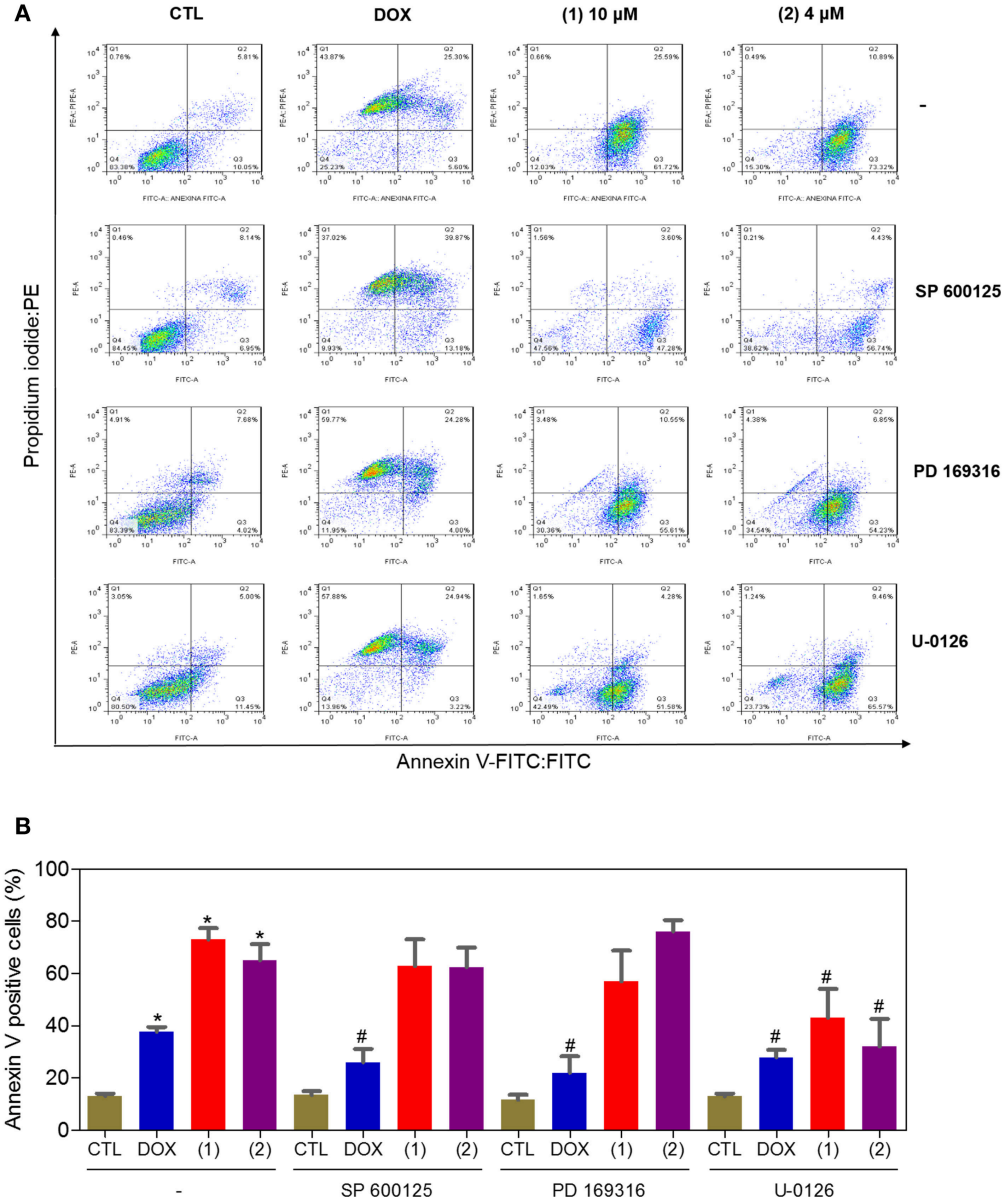
Effect of the JNK/SAPK (SP600125), p38 MAPK (PD169316), and MEK (U0126) inhibitors on the apoptosis that was induced by ruthenium complexes containing heterocyclic thioamidates in HepG2 cells, as determined by flow cytometry using annexin V-FITC/PI staining. **(A)** Representative flow cytometry dot plots showing the percentage of cells in the viable, early-apoptotic, late-apoptotic and necrotic stages. **(B)** Quantification of the apoptotic cells (early- and late-apoptotic cells). For the protection assays, the cells were preincubated for 2 h with 5 μM SP600125, 5 μM PD169316 or 5 μM U0126 and were then incubated with 10 μM of complex **1** or 4 μM of complex **2** for 48 h. The negative control (CTL) was treated with the vehicle (0.2% DMSO) that was used to solubilize and dilute the complexes, and doxorubicin (DOX, 2 μM) was used as the positive control. Data are presented as the mean ± S.E.M. of three independent experiments that were performed in duplicate. *P < 0.05 compared with the negative control, as determined by ANOVA followed by the Student-Newman-Keuls test. #*P* < 0.05 compared with the respective treatment without inhibitor, as determined by ANOVA followed by the Student-Newman-Keuls test. Ten thousand events were evaluated per experiment, and cellular debris was omitted from the analysis.

**Figure 11:**
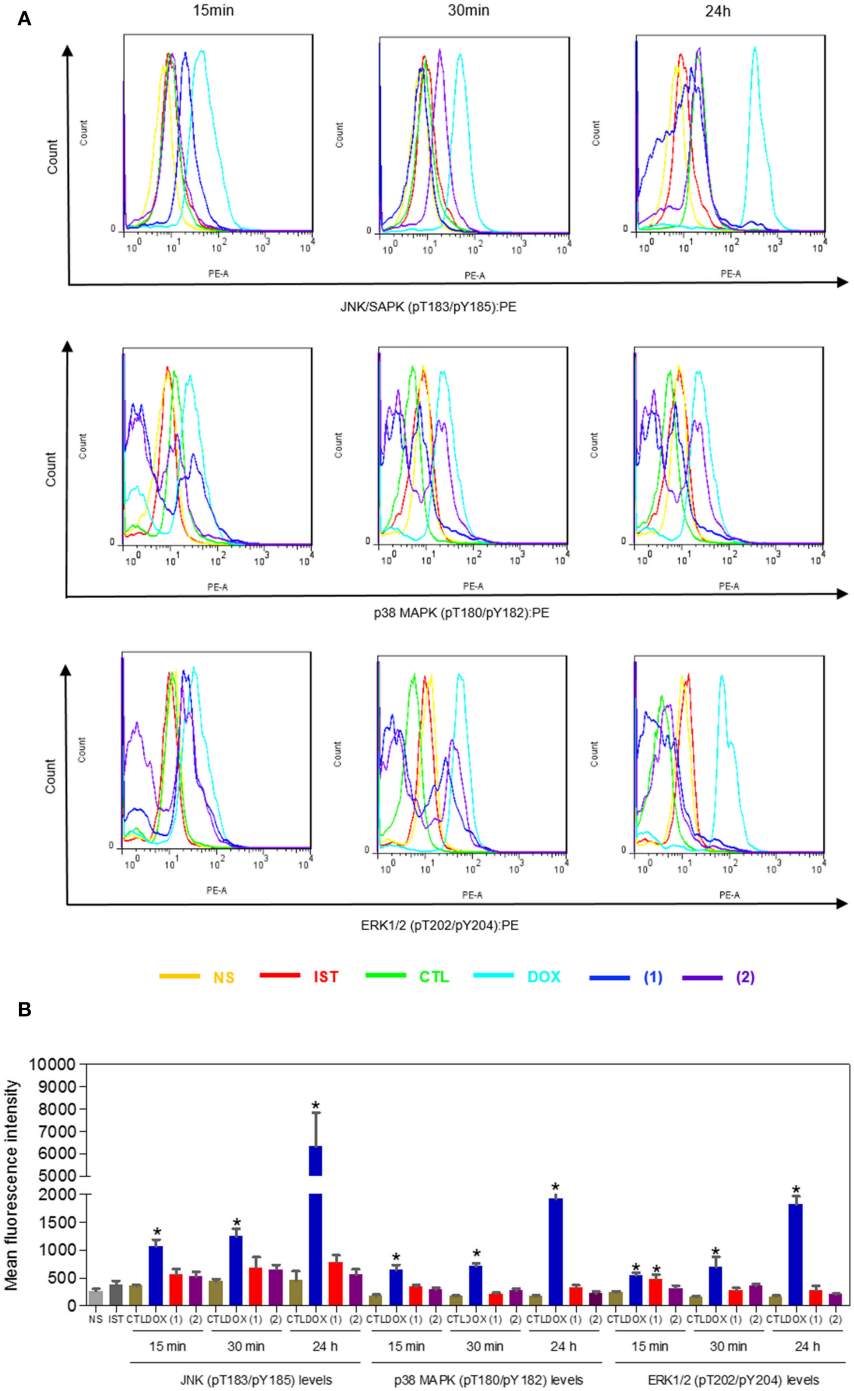
Effect of ruthenium complexes containing heterocyclic thioamidates on the JNK/SAPK (pT183/pY185), p38 MAPK (pT180/pY182), and ERK1/2 (pT202/pY204) levels, as determined by the phosflow analysis of HepG2 cells treated with 10 μM of complex **1** or 4 μM of complex **2** for an acute (15 or 30 min) or prolonged (24 h) incubation. **(A)** Representative flow cytometry histograms. **(B)** Quantification of the JNK/SAPK (pT183/pY185), p38 MAPK (pT180/pY182) and ERK1/2 (pT202/pY204) levels. The negative control (CTL) was treated with the vehicle (0.2% DMSO) that was used to solubilize and dilute the complexes, and doxorubicin (DOX, 2 μM) was used as the positive control. Data are presented as the mean ± S.E.M. of three independent experiments that were performed in duplicate. *P < 0.05 compared with the negative control, as determined by ANOVA followed by the Student-Newman-Keuls test. Ten thousand events were evaluated per experiment, and cellular debris was omitted from the analysis. NS, non-stained cells (basal cell fluorescence); IST, isotype control.

Since mitogen-activated protein kinase (MAPK) signaling plays a crucial role in apoptosis that is induced by DNA intercalating agents, we decided to assess the role of the three MAPK proteins, ERK1/2, JNK/SAPK and p38 MAPK, in apoptosis that is induced by ruthenium complexes containing heterocyclic thioamidates in HepG2 cells. Initially, we quantified apoptosis induced by the complexes in HepG2 cells that were coincubated with a JNK/SAPK inhibitor (SP600125), p38 MAPK inhibitor (PD169316), and an MEK inhibitor (U-0126, which inhibits the activation of ERK1/2) (Figure 10). Next, we quantified the phosphorylation status of JNK/SAPK (pT183/pY185), p38 MAPK (pT180/pY182) and ERK1/2 (pT202/pY204) expression in HepG2 cells after acute (15 and 30 min) and prolonged (24 h) incubations with the complexes (Figure 11). Compared to the negative controls, complex **1** induced the phosphorylation of ERK1/2 after 15 min of incubation, but no significant increase was observed in the phosphorylation of JNK/SAPK or p38 MAPK. Furthermore, coincubation with an MEK inhibitor reduced the level of apoptosis that was induced by both complexes compared to negative controls. Coincubation with a JNK/SAPK inhibitor or a p38 MAPK inhibitor did not reduce the level of apoptosis induced by these complexes compared to that in the negative controls.

Moreover, ERK1/2 activation is associated with the induction of oxidative stress and/or the activation of p53 signaling. Thus, we assessed the role of oxidative stress induction and p53 activation in apoptosis by the complexes in HepG2 cells. First, we quantified ROS levels in HepG2 cells treated with the complexes for 1 and 3 h (Figure S1). Then, we quantified the level of apoptosis induced by the complexes in HepG2 cells that were coincubated with an antioxidant (NAC) (Figure S2) and a p53 inhibitor (cyclic pifithrin-α) (Figure S3). However, compared to the negative controls, the complexes did not induce a significant increase in ROS levels, and coincubation with an antioxidant and a p53 inhibitor did not reduce the apoptosis induced by the complexes, indicating that the complexes induce apoptosis through ROS- and p53-independent pathways in HepG2 cells.

### Ruthenium Complexes Containing Heterocyclic Thioamidates Reduce HepG2 Cell Growth in a Xenograft Model

**Figure 12:**
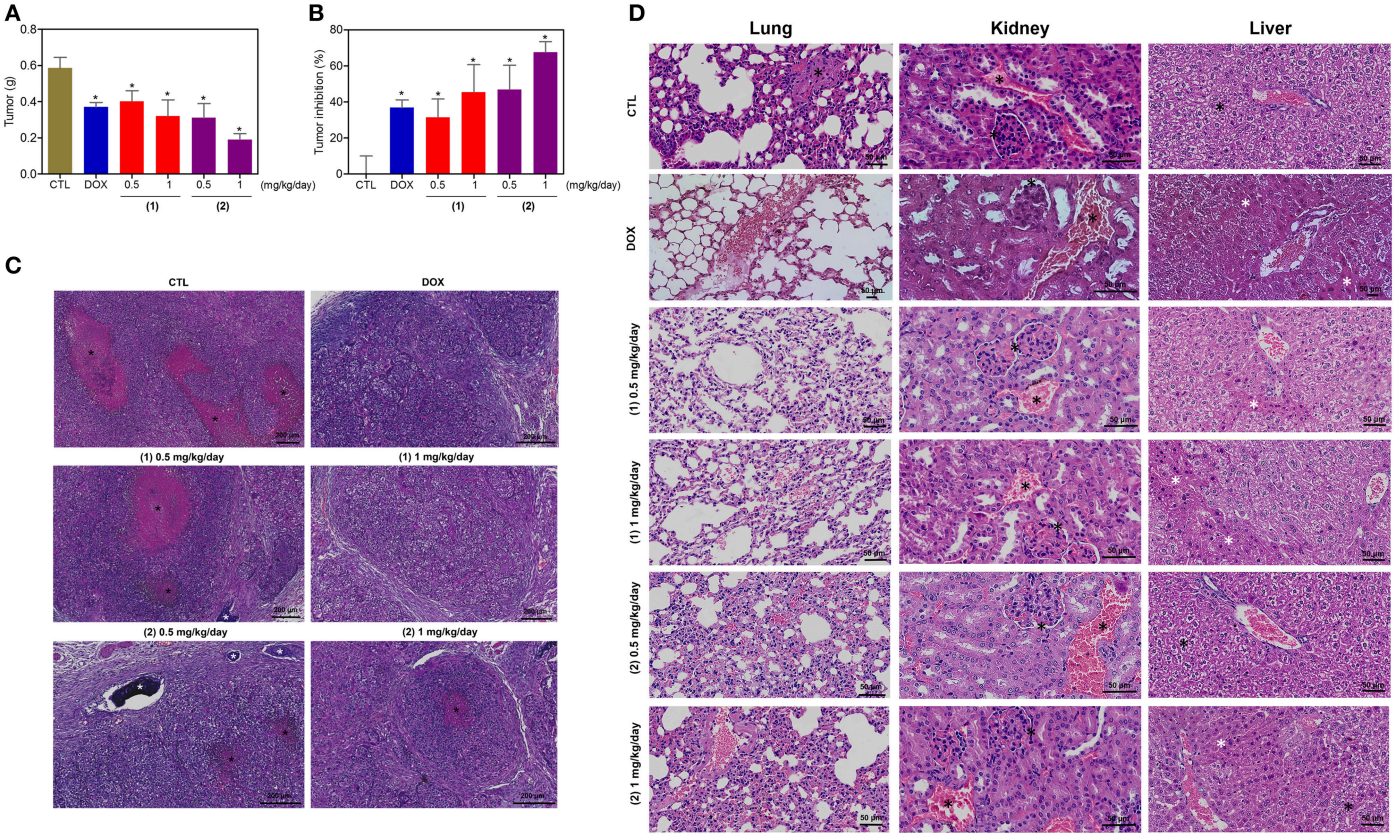
*In vivo* antitumor activity of ruthenium complexes containing heterocyclic thioamidates in C.B-17 SCID mice with HepG2 cell xenografts. **(A)** Quantification of the tumor weight (g). **(B)** Quantification of the tumor inhibition (%). Data are presented as the mean ± S.E.M. of 8–10 animals. *P < 0.05 compared with the negative control, as determined by ANOVA followed by the Student-Newman-Keuls test. **(C)** Representative histological analysis of the tumors that were stained with hematoxylin and eosin and analyzed by light microscopy. The areas of necrosis and dystrophic calcification are represented by black and white asterisks, respectively. **(D)** Representative histological analysis of the lungs, kidneys and livers that were stained with hematoxylin and eosin and analyzed by light microscopy. Black asterisks represent metastasis (lung), the thickening of the basal membrane of the renal glomerulus with decreased urinary space or vascular congestion (kidney) and hydropic degeneration (liver). White asterisks represent the coagulation necrosis of hepatic cells. The negative control (CTL) was treated with the vehicle (5% DMSO) that was used to solubilize and dilute the complexes, and doxorubicin (DOX, 0.3 mg/kg/day) was used as the positive control. Beginning 1 day after tumor implantation, the animals were treated via an intraperitoneal route for 21 consecutive days.

The *in vivo* anti-liver cancer activity of ruthenium complexes containing heterocyclic thioamidates was assessed in C.B-17 SCID mice engrafted with HepG2 cells. The animals were treated with the complexes at doses of 0.5 and 1 mg/kg/day by intraperitoneal injections for 21 consecutive days. Both complexes were able to inhibit HepG2 cell growth in mice compared to that of the negative controls (Figures 12A,B). At the end of the treatment, the mean tumor mass of the negative control animals was 0.6 ± 0.1 g. In the animals treated with complex **1**, the mean tumor masses were 0.4 ± 0.1 g and 0.3 ± 0.1 g at the lower and higher doses, respectively. In the animals treated with complex **2**, the mean tumor masses were 0.3 ± 0.1 g and 0.2 ± 0.1 g at the lower and higher doses, respectively. Compared to the negative controls, the tumor mass inhibition rates were 31.5–45.4% and 46.9–67.7% for complexes **1** and **2**, respectively. Doxorubicin (at a dose of 0.3 mg/kg/day) reduced the tumor weight by 36.8% compared to that of the negative controls. In the histological analysis, all groups exhibited a solid pattern composed of polygonal and pleomorphic tumor cells, eosinophilic granular cytoplasm, rounded nuclei and prominent nucleoli (Figure 12C). The stroma was fibrous and hypervascular. The group of animals in the negative control group exhibited larger tumors, and the areas of necrosis, dystrophic calcification and inflammation were more evident than those in the other experimental groups. For the doxorubicin group and the groups that were treated with complexes **1** and **2**, the tumors were more encapsulated, and the tumor cell islands were smaller, with fewer mitotic figures when compared to those in the negative control group. The most evident fibrosis was observed in the complex **2** group (1 mg/kg/day) compared to that in the other groups.

Some toxicological aspects were also evaluated in the mice treated with the complexes. There was no significant alteration in the body weight of animals treated with the complexes (*P* > 0.05) compared to that in the negative controls. No significant alterations were observed in the liver, kidney, lung or heart wet weight of any group (*P* > 0.05) (Table S2). In addition, the number of white and erythrocyte blood cells in the mice treated with the complexes was also examined (Table S3). The number of leukocytes and erythrocytes remained unchanged after treatment with the complexes compared to that in the controls (*P* > 0.05).

Morphological analyses of the liver, kidneys, lungs, and heart in all groups were performed (Figure 12D). In the liver, the acinar architecture and centrilobular vein were preserved in all groups. The inflammation observed in the liver portal space was mild in most animals. Other findings, such as congestion and hydropic degeneration, were found in all groups, ranging from mild to moderate. In addition, focal areas of coagulation necrosis were observed in all groups and were more evident in the complex **2** group compared to the other groups, indicating hepatotoxicity. In the kidneys, the tissue architecture was maintained in all experimental groups. Histopathological changes, including vascular congestion and the thickening of the basal membrane of the renal glomerulus with decreased urinary space, were observed in all kidneys. In the lungs, the architecture of the parenchyma was partially maintained in all groups, and a thickening of the alveolar septum with decreased airspace was observed, ranging from mild to moderate. In addition, some animals in the experimental groups showed mild emphysema. Significant inflammation, predominantly of mononuclear cells, edema, congestion and hemorrhage were frequently observed, ranging from mild to severe. Tumor nodules in the lungs were observed only in one animal in the negative control group (DMSO 5%). The heart did not show alterations in any group.

## Discussion

The current prospects for the use of ruthenium complexes as potential antineoplastic drugs is both promising, particularly for some specific candidates, as well as disappointing, since none of these molecules has been approved for clinical use in cancer therapy at the present moment (27, 28). The fact that almost 30 years after the first studies with ruthenium complexes were performed, these compounds continue to be viewed as only “promising compounds” is worrisome (27). On the other hand, an impressive number of studies have demonstrated the potential of ruthenium complexes as next-generation anticancer metallotherapeutics that show more selectivity to cancer cells and the ability to overcome the resistance that is observed for platinum-based drugs (28). In this paper, a series of ruthenium complexes containing heterocyclic thioamidates were studied as novel anticancer drug candidates for liver cancer treatment.

Herein, we demonstrated for the first time that the ruthenium complexes containing heterocyclic thioamidates that were studied in this work bind to DNA, inhibit cell proliferation, trigger caspase-mediated apoptosis through ERK1/2 signaling in HepG2 cells, and can reduce the growth of HepG2 cells that are engrafted in C.B-17 SCID mice. The ruthenium complexes containing heterocyclic thioamidates that were tested in this work had potent cytotoxicity against cancer cells with different histological types, with IC_50_ values below 20 μM, and complexes **1** and **2** were the most potent cytotoxic agents, with IC_50_ values below 10 μM. These data corroborate the results of Correa et al. (18) and Silva (19), who reported that when these complexes were tested in a small panel of cancer cells (MCF-7, DU-145 [human prostate carcinoma], A549 [human lung carcinoma], and HepG2), they had IC_50_ values below 10 μM.

Various metal-based complexes have been synthesized containing heterocyclic thioamidates with cytotoxic properties. A silver-chloride complex with the heterocyclic thioamide 5-chloro-2-mercaptobenzothiazole and tri(p-tolyl)phosphine displayed cytotoxicity to leiomyosarcoma cancer cells (LMS) from Wistar rats, with an IC_50_ value of 13.7 μM (29). On the other hand, different metallocomplexes have been generated using heterocyclic thioamidates that were only cytotoxic at high concentrations. Ruthenium complexes containing the thioamide ligand (2-hydroxy-5-nitrophenyl)(pyrrolidin-1-yl)methanethione were previously synthesized and investigated for their cytotoxic activity, showing IC_50_ values above 40 μM in human cervical carcinoma (HeLa) and breast adenocarcinoma (MCF-7) cell lines (30). The bismuth complex with the heterocyclic thioamide 2-mercapto-1-methylimidazole had IC_50_ values above 30 μM for the HeLa and MCF-7 cell lines (31). The antimony bromide complex with the heterocyclic thioamide 2-mercapto-1-methylimidazole had IC_50_ values above 15 μM in murine leukemia cells (L1210), murine mammary carcinoma cells (FM3A), human T-lymphocyte (Molt4/C8, CEM), and human cervix carcinoma cells (HeLa)(32). In our outcomes, ruthenium-based heterocyclic thioamidates displayed greater cytotoxicity then previous heterocyclic thioamidates metallocomplexes.

As mentioned above, the ruthenium complexes containing heterocyclic thioamidates that were studied in this work were previously reported to bind to DNA and bovine serum albumin and to inhibit DNA supercoiled relaxation that is mediated by human topoisomerase IB (18, 19). Herein, we observed that these ruthenium complexes behave like DNA intercalating agents, which can be caused by drug-DNA interactions at the minor groove or/and electrostatic interactions. These effects can occur with octahedral compounds, such as the ruthenium complexes, that are very bulky and that do not intercalate (33, 34). A ruthenium complex containing guanidine has been reported to bind to DNA and induce DNA damage, cell cycle arrest and to activate the typical apoptosis pathways in MCF-7 cells (35). A ruthenium complex with thymine was reported to bind to DNA and human and bovine serum albumin and to induce caspase-mediated apoptosis in human promyelocytic leukemia HL-60 cells (9, 10). On the other hand, the half-sandwich iridium and ruthenium complexes containing P^∧^P-chelating ligands cause cell apoptosis in human lung carcinoma A549 cells, and although these types of complexes interact with ctDNA, DNA appears not to be the major target (36). Moreover, the ruthenium complexes with 5-fluorouracil display enhanced cytotoxicity to different cancer cells and are able to induce caspase-mediated apoptosis in HCT116 cells but do not induce DNA intercalation (11). In the present work, we observed that ruthenium complexes containing heterocyclic thioamidates bind to DNA and inhibit cell proliferation, triggering caspase-mediated apoptosis in HepG2 cells.

Additionally, we investigated the role of the MAPK pathway in apoptosis that is induced by ruthenium complexes containing heterocyclic thioamide. MAPKs are serine/threonine protein kinases that have an important function in both cell survival and cell death. The MAPK pathway is currently known to comprise four subpathways: ERK (ERK1 and ERK2), JNK/SAPK (JNK1, JNK2, and JNK3), p38 MAPK (α, β, δ, and γ) and Big MAP kinase 1 (BMK1 or ERK5). The effects of the activation of ERK1/2 include gene expression that promotes cell proliferation, differentiation and cellular survival. The functions of JNK/SAPK and p38 MAPK activation include cell differentiation, apoptosis and survival. However, the function and regulation of BMK1/ERK5 have been less intensively explored, and it has been reported that this subpathway can regulate cell growth, differentiation and survival (37–41).

Herein, we observed that ruthenium complexes containing heterocyclic thioamidates induce apoptosis that is mediated by MAPK ERK1/2 signaling. In fact, although the activation of ERK1/2 has mainly been associated with a prosurvival function, DNA damage-induced ERK1/2 activation causes apoptotic cell death (12, 42–44). The induction of ERK1/2-mediated apoptosis by the ruthenium complexes containing heterocyclic thioamidates that were studied in this work seems to occur via direct DNA damage since these complexes failed to induce ROS production (an indirect mechanism of DNA damage induction) and were able to induce DNA intercalation. Moreover, ERK1/2 activation can induce apoptosis via a p53-dependent or p53-independent pathway (43, 45, 46). The apoptosis induced by the ruthenium complexes containing heterocyclic thioamidates was not reduced by coincubation with a p53 inhibitor, indicating that these complexes cause apoptosis through a p53-independent pathway in HepG2 cells. In support of these data, previous reports have also shown ruthenium-based compounds to induce apoptosis in cancer cells via MAPK pathways that were either ROS- and p53-dependent or independent (12, 47, 48).

In addition, we observed that the ruthenium complexes containing heterocyclic thioamidates reduced the growth of HepG2 cells that were engrafted in C.B-17 SCID mice and had a similar efficacy as the positive control doxorubicin. In relation to toxicity, evidence of hepatotoxicity was observed after treatment with complex **2**. On the other hand, although some histological changes were found, these changes may be reversed after treatment. Similarly, ruthenium imidazole complex and association of ruthenium-arene complex and erlotinib were also able to inhibit cancer cell growth in mice engrafted with A549 and A2780 cells, respectively (49, 50).

**Figure 13:**
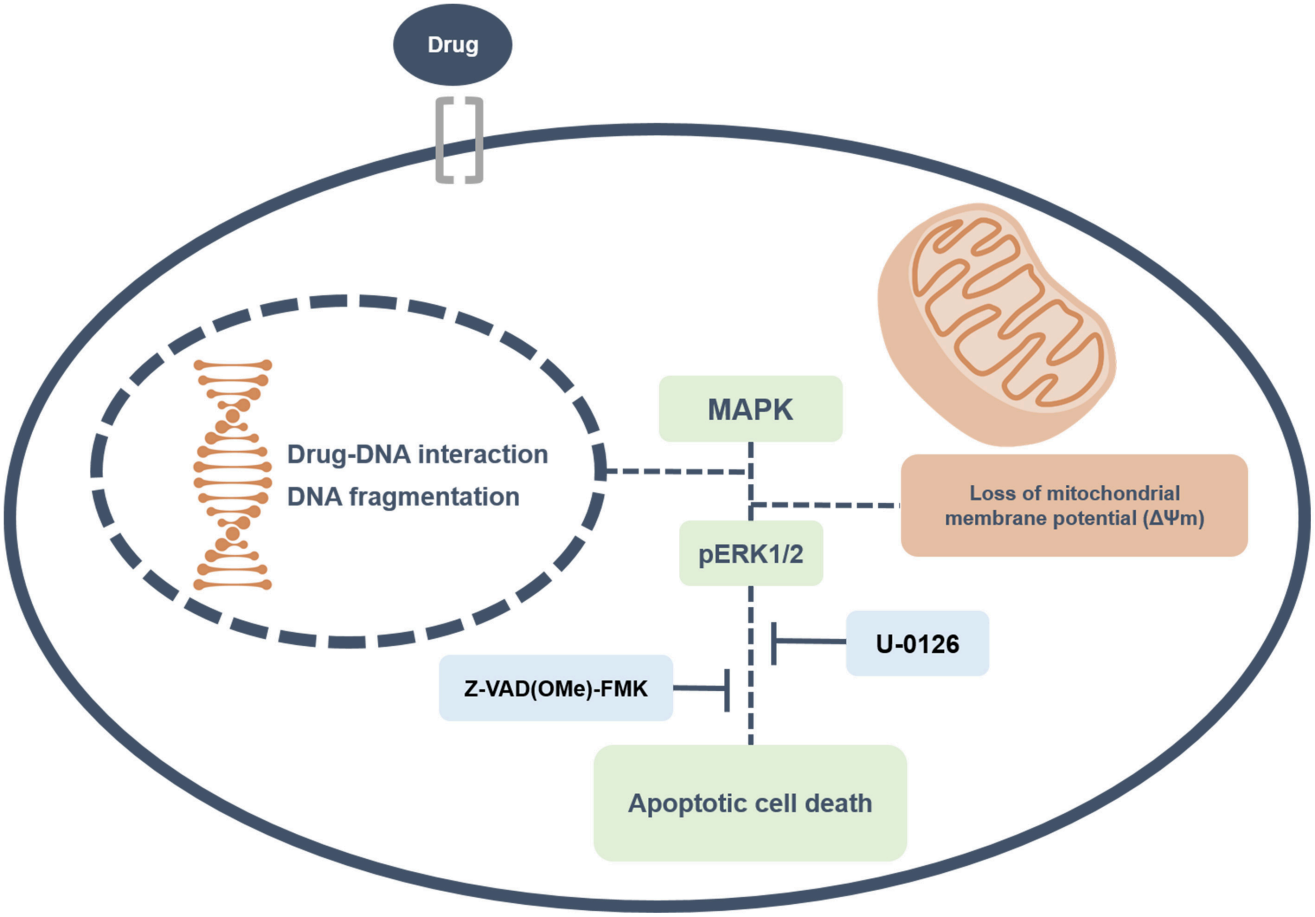
Summary of the molecular mechanisms of ruthenium complexes containing heterocyclic thioamidates in HepG2 cells. Herein, we revealed that these complexes bind to DNA triggering caspase-mediated apoptosis through ERK1/2 signaling in HepG2 cells.

Overall, this study revealed that ruthenium complexes containing heterocyclic thioamidates bind to DNA and inhibit cell proliferation, triggering caspase-mediated apoptosis through ERK1/2 signaling in HepG2 cells, which occurs via a ROS- and p53-independent pathway (Figure 13). Moreover, these complexes reduce the growth of HepG2 cells that are engrafted in C.B-17 SCID mice. These results indicate that these complexes are novel anticancer drug candidates for liver cancer treatment.

## Data Availability

The raw data supporting the conclusions of this manuscript will be made available by the authors, without undue reservation, to any qualified researcher.

## Ethics Statement

PBMC were obtained from peripheral blood of donors voluntary with informed consent (number 031019/2013) approved by the Human Ethics Committee of Gonçalo Moniz Institute from Oswaldo Cruz Foundation (IGM-FIOCRUZ/BA). The animal experiments were conducted in accordance with the experimental protocol (number 06/2015) approved by the Animal Ethics Committee of IGM-FIOCRUZ/BA.

## Author Contributions

SN, MdS, MS, CR, AB, and DB conceived and designed the experiments. SN, NdC, AR, and LB performed the *in vitro* and *in vivo* experiments. SN, NdC, MdS, AR, LB, RD, CS, CR, and DB analyzed the data. CR, MS, AB, and DB contributed reagents, materials, and analysis tools. DB wrote the paper. All authors read and approved the final manuscript.

### Conflict of Interest Statement

The authors declare that the research was conducted in the absence of any commercial or financial relationships that could be construed as a potential conflict of interest.

## Abbreviations

ANOVA: analysis of variance
ATCC: American type culture collection
BAD KO SV40 MEF: BAD gene knockout immortalized mouse embryonic fibroblast
bipy: 2,2′-bipyridine
ctDNA: calf thymus DNA
DCF-DA, 2′: 7′-dichlorofluorescin diacetate
dmp: 4,6-diamino-2-mercaptopyrimidine
DMSO: dimethyl sulfoxide
dppb: 1,4-bis(diphenylphosphino)butane
ERK: extracellular signal-regulated kinase
HCC: hepatocellular carcinoma
IC_50_: half-maximal inhibitory concentration
Im: imidazole
Ind: indazole
JNK/SAPK: Jun kinase
KP1019: IndH]trans-[RuCl_4_(Ind)_2_]
MAPKs: mitogen-activated protein kinases
mmi: mercapto-1-methyl-imidazole
mpca: 6-mercaptopyridine-3-carboxylic acid
NAC: *N*-acetylcysteine
NAMI-A: ImH]trans-[RuCl_4_(Im)(dmso-S)
PBMCs: peripheral blood mononuclear cells
ROS: reactive oxygen species
SCID: severe-combined immunodeficiency
SI: selectivity index
TBE: trypan blue exclusion
tzdt: 1,3-thiazolidine-2-thione
WT SV40 MEF: wild-type immortalized mouse embryonic fibroblast.
