# Supplementary material for: Ruthenium Complexes Containing Heterocyclic Thioamidates Trigger Caspase-Mediated Apoptosis Through MAPK Signaling in Human Hepatocellular Carcinoma Cells
Source: Front Oncol. 2019 Jul 9;9:562. doi: 10.3389/fonc.2019.00562 (PMC6629894; doi:10.3389/fonc.2019.00562)
Supplement: Supplementary file 1 [file Data_Sheet_1.doc]

**Supplementary Material**

Ruthenium complexes containing heterocyclic thioamidates trigger caspase-mediated apoptosis through MAPK signaling in human hepatocellular carcinoma cells

Sara P. Neves^1^, Nanashara C. de Carvalho^1^, Monize M. da Silva^2^, Ana Carolina B. C. Rodrigues^1^, Larissa M. Bomfim^1^, Rosane B. Dias^1^, Caroline B. S. Sales^3^, Clarissa A. G. Rocha^1^, Milena B. P. Soares^1^, Alzir A. Batista^3^, Daniel P. Bezerra^1^,*

^1^Gonçalo Moniz Institute, Oswaldo Cruz Foundation (IGM-FIOCRUZ/BA), Salvador, Bahia, Brazil.

^2^Department of Chemistry, Federal University of São Carlos, São Carlos, São Paulo, 13561-901, Brazil.

^3^Department of Biomorphology, Institute of Health Sciences, Federal University of Bahia, Salvador, Bahia, 40110-902, Brazil.

*** Corresponding author**

Prof. Dr. Daniel P. Bezerra, Gonçalo Moniz Institute, Oswaldo Cruz Foundation (IGM-FIOCRUZ/BA), Rua Waldemar Falcão, 121, Candeal, 40296-710, Salvador, Bahia, Brazil. E-mail: danielpbezerra@gmail.com Tel/Fax + 55 71 3176 2272

**Figure S1.** Effect of ruthenium complexes containing heterocyclic thioamidates in the levels of reactive oxygen species (ROS) of HepG2 cells after 1 and 3 h of incubation, as determined by flow cytometry using DCF-DA staining. The negative control (CTL) was treated with the vehicle (0.2% DMSO) that was used to solubilize and dilute the complexes, and hydrogen peroxide (H_2_O_2_, 200 µM) and doxorubicin (DOX, 2 µM) were used as the positive controls. Data are presented as the mean ± S.E.M. of three independent experiments that were performed in duplicate. * *P* < 0.05 compared with the negative control, as determined with ANOVA followed by the Student-Newman-Keuls test. Ten thousand events were evaluated per experiment, and cellular debris was omitted from the analysis. MFI: mean fluorescence intensity.


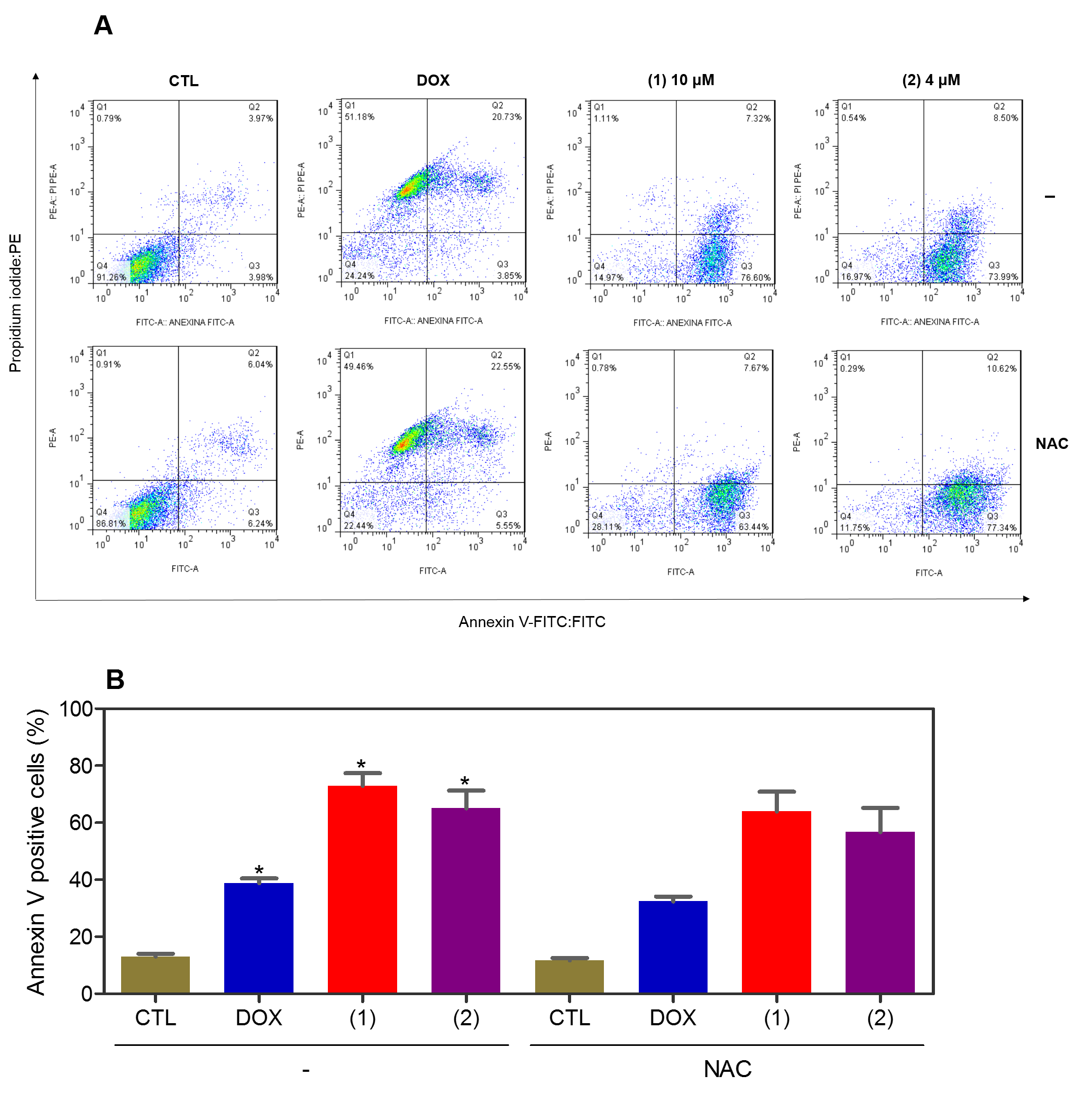


**Figure S2.** Effect of antioxidant N-acetylcysteine (NAC) on the apoptosis that was induced by ruthenium complexes containing heterocyclic thioamidates in HepG2 cells, as determined by flow cytometry using annexin V-FITC/PI staining. (**A**) Representative flow cytometry dot plots showing the percentage of cells in the viable, early-apoptotic, late-apoptotic and necrotic stages. (**B**) Quantification of the apoptotic cells (early- and late-apoptotic cells). For the protection assay, the cells were preincubated for 2 h with 5 mM NAC and were then incubated with 10 µM of complex **1** or 4 µM of complex **2** for 48 h. The negative control (CTL) was treated with the vehicle (0.2% DMSO) that was used to solubilize and dilute the complexes, and doxorubicin (DOX, 2 µM) was used as the positive control. Data are presented as the mean ± S.E.M. of three independent experiments that were performed in duplicate. * *P* < 0.05 compared with the negative control, as determined by ANOVA followed by the Student-Newman-Keuls test. Ten thousand events were evaluated per experiment, and cellular debris was omitted from the analysis.


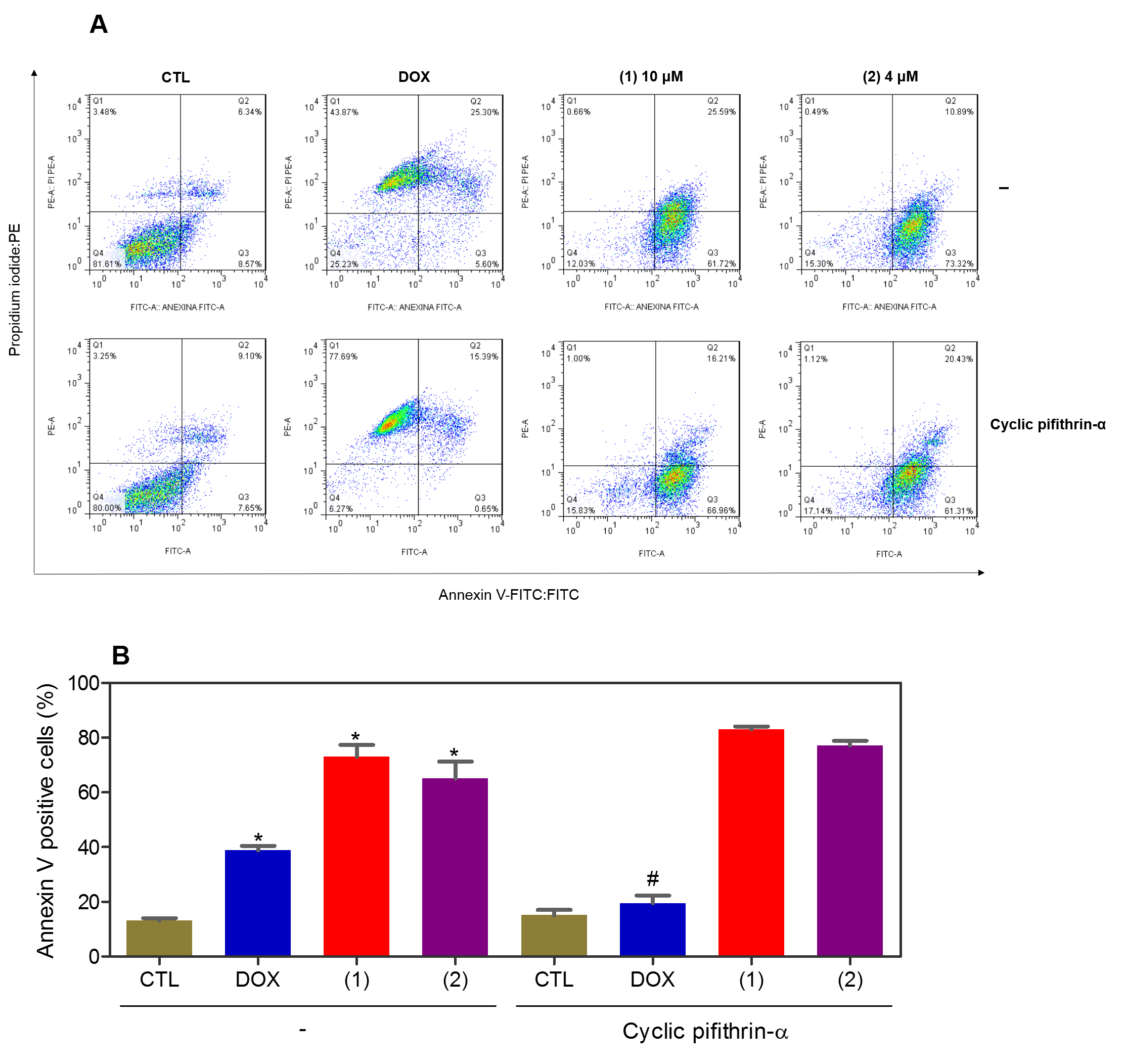


**Figure S3.** Effect of the p53 inhibitor (cyclic pifithrin-α) on the apoptosis that was induced by ruthenium complexes containing heterocyclic thioamidates on HepG2 cells, as determined by flow cytometry using annexin V-FITC/PI staining. (**A**) Representative flow cytometry dot plots showing the percentage of cells in the viable, early-apoptotic, late-apoptotic and necrotic stages. (**B**) Quantification of the apoptotic cells (early- and late-apoptotic cells). For the protection assay, the cells were preincubated for 2 h with 10 µM cyclic pifithrin-α and were than incubated with 10 µM of complex **1** or 4 µM of complex **2** for 48 h. The negative control (CTL) was treated with the vehicle (0.2% DMSO) that was used to solubilize and dilute the complexes, and doxorubicin (DOX, 2 µM) was used as the positive control. Data are presented as the mean ± S.E.M. of three independent experiments that was performed in duplicate. * *P* < 0.05 compared with the negative control, as determined by ANOVA followed by the Student-Newman-Keuls test. # *P* < 0.05 compared with the respective treatment without inhibitor, as determined by ANOVA followed by the Student-Newman-Keuls test. Ten thousand events were evaluated per experiment and cellular debris was omitted from the analysis.

**Table S1.** Cytotoxic activity of ruthenium complexes containing heterocyclic thioamidates in WT SV40 MEF and BAD KO SV40 MEF cell lines

| **Drugs** | **IC_50_ and 95% CI (μM)** | |
| --- | --- | --- |
|  | **WT SV40 MEF** | **BAD KO SV40 MEF** |
| **(1)** | 1.1  0.9 – 1.2 | 0.7  0.4 - 1.1 |
| **(2)** | 2.0  1.7 – 2.4 | 2.9  1.5 – 5.7 |
| DOX | 0.04  0.02 - 0.22 | 0.4  0.2 – 0.9 |
| CDDP | 36.9  23.9 – 57.0 | 47.3  37.2 – 60.1 |

Data are presented as the IC_50_ values and 95% confidence intervals (95% CI) in µM that were obtained with nonlinear regression from at least three independent experiments that were performed in duplicate, as measured by an Alamar blue assay after 72 h of incubation. Cell lines: WT SV40 MEF (wild-type immortalized mouse embryonic fibroblasts); and BAD KO SV40 MEF (BAD gene knockout immortalized mouse embryonic fibroblasts). Doxorubicin (DOX) and cisplatin (CDDP) were used as the positive controls.

**Table S2.** Effect of ruthenium complexes containing heterocyclic thioamidates on body and relative organ weight from C.B-17 SCID mice engrafted with HepG2 cells

| **Parameters** | **CTL** | **DOX** | **(1)** | **(1)** | **(2)** | **(2)** |
| --- | --- | --- | --- | --- | --- | --- |
| Dose (mg/kg/day) | - | 0.3 | 0.5 | 1 | 0.5 | 1 |
| Survival | 10/10 | 14/14 | 10/10 | 10/10 | 10/10 | 10/10 |
| Initial body weight (g) | 20.3 ± 0.7 | 22.3 ± 0.4 | 21.7 ± 0.2 | 20.3 ± 0.2 | 20.4 ± 0.4 | 19.9 ± 0.5 |
| Final body weight (g) | 20.5 ± 0.4 | 19.5 ± 0.6 | 20.4 ± 0.3 | 20.9 ± 0.4 | 20.8 ± 0.4 | 19.4 ± 0.5 |
| Liver (g/100 g body weight) | 5.3 ± 0.2 | 4.8 ± 0.1 | 4.7 ± 0.2 | 4.9 ± 0.2 | 4.7 ± 0.2 | 4.6 ± 0.1 |
| Kidney (g/100 g body weight) | 1.6 ± 0.1 | 1.5 ± 0.04 | 1.6 ± 0.1 | 1.6 ± 0.1 | 1.5 ± 0.1 | 1.5 ± 0.1 |
| Heart (g/100 g body weight) | 0.6 ± 0.1 | 0.5 ± 0.02 | 0.5 ± 0.1 | 0.6 ± 0.1 | 0.6 ± 0.1 | 0.6 ± 0.1 |
| Lung (g/100 g body weight) | 0.7 ± 0.1 | 0.9 ± 0.06 | 0.7 ± 0.1 | 0.7 ± 0.1 | 0.8 ± 0.1 | 0.8 ± 0.1 |

The negative control (CTL) was treated with the vehicle (5% DMSO) that was used to solubilize and dilute the complexes, and doxorubicin (DOX) was used as the positive control. Beginning 1 day after tumor implantation, the animals were treated through the intraperitoneal route for 21 consecutive days. Data are presented as the mean ± S.E.M. of 8-10 animals.

**Table S3.** Effect of ruthenium complexes containing heterocyclic thioamidates on hematological parameters of peripheral blood from C.B-17 SCID mice engrafted with HepG2 cells

| **Parameters** | **CTL** | **DOX** | **(1)** | **(1)** | **(2)** | **(2)** |
| --- | --- | --- | --- | --- | --- | --- |
| Dose (mg/kg/day) | - | 0.3 | 0.5 | 1 | 0.5 | 1 |
| Erythrocytes  (10^6^cells/μL) | 9.0 ± 0.8 | 8.9 ± 0.7 | 10.5 ± 0.6 | 6.7 ± 0.9 | 8.5 ± 1.0 | 7.8 ± 0.8 |
| Total leukocytes (10^3^cells/μL) | 3.5 ± 0.5 | 1.5 ± 0.2* | 2.3 ± 0.4 | 3.9 ± 1.9 | 2.5 ± 0.4 | 2.7 ± 0.4 |
| Differential leukocytes (%) | |  |  |  |  |  |
| Neutrophils | 14.6 | 48.8 | 23.8 | 24.4 | 21.1 | 34.4 |
| Lymphocytes | 47.0 | 49.3 | 36.4 | 55.5 | 39.9 | 32.2 |
| Monocytes | 38.5 | 1.0 | 39.9 | 20.1 | 39.1 | 32.5 |
| Eosinophils | 0.3 | 1.0 | 0.7 | 1.4 | 1.3 | 1.1 |

The negative control (CTL) was treated with the vehicle (5% DMSO) that was used to solubilize and dilute the complexes, and doxorubicin (DOX) was used as the positive control. Beginning 1 day after tumor implantation, the animals were treated through the intraperitoneal route for 21 consecutive days. Data are presented as the mean ± S.E.M. of 4-6 animals. * *P* < 0.05 compared with the negative control as determined by ANOVA, followed by the Student-Newman-Keuls Test.
